# Molecular characterization of respiratory syncytial viruses circulating in a paediatric cohort in Amman, Jordan

**DOI:** 10.1099/mgen.0.000292

**Published:** 2019-09-18

**Authors:** Nídia S. Trovão, Najwa Khuri-Bulos, Yi Tan, Vinita Puri, Meghan H. Shilts, Rebecca A. Halpin, Nadia B. Fedorova, Martha I. Nelson, Natasha Halasa, Suman R. Das

**Affiliations:** ^1^​ Division of International Epidemiology and Population Studies, Fogarty International Center, National Institutes of Health, Bethesda, MD, USA; ^2^​ Division of Infectious Diseases, Department of Pediatrics, University of Jordan, Amman, Jordan; ^3^​ Department of Medicine, Vanderbilt University Medical Center, Nashville, TN, USA; ^4^​ Infectious Disease Group, J. Craig Venter Institute, Rockville, MD, USA; ^5^​ Departments of Pediatrics, Vanderbilt University, Nashville, TN, USA

**Keywords:** respiratory syncytial virus, paediatric cohort, next-generation sequencing, evolution, phylogenetic analysis, Bayesian analysis

## Abstract

Respiratory syncytial viruses (RSVs) are an important cause of mortality worldwide and a major cause of respiratory tract infections in children, driving development of vaccine candidates. However, there are large gaps in our knowledge of the local evolutionary and transmission dynamics of RSVs, particularly in understudied regions such as the Middle East. To address this gap, we sequenced the complete genomes of 58 RSVA and 27 RSVB samples collected in a paediatric cohort in Amman, Jordan, between 2010 and 2013. RSVA and RSVB co-circulated during each winter epidemic of RSV in Amman, and each epidemic comprised multiple independent viral introductions of RSVA and RSVB. However, RSVA and RSVB alternated in dominance across years, potential evidence of immunological interactions. Children infected with RSVA tended to be older than RSVB-infected children [30 months versus 22.4 months, respectively (*P* value = 0.02)], and tended to developed bronchopneumonia less frequently than those with RSVB, although the difference was not statistically significant (*P* value = 0.06). Differences in spatial patterns were investigated, and RSVA lineages were often identified in multiple regions in Amman, whereas RSVB introductions did not spread beyond a single region of the city, although these findings were based on small sample sizes. Multiple RSVA genotypes were identified in Amman, including GA2 viruses as well as three viruses from the ON1 sub-genotype that emerged in 2009 and are now the dominant genotype circulating worldwide. As vaccine development advances, further sequencing of RSV is needed to understand viral ecology and transmission, particularly in under-studied locations.

## Data Summary

All accession numbers of the genetic sequences used in this study are listed in the Supplementary material (available with the online version of this article).

**Impact Statement**

With multiple respiratory syncytial virus (RSV) vaccine candidates in development, it is crucial to understand the evolutionary dynamics of the virus, including in regions where there is little data available, such as the Middle East. By investigating RSV in a paediatric cohort in Amman, Jordan, we were able to study the population dynamics of the virus over time, including the alternating dominance of the RSVA and RSVB subgroups, the association between viral genetics and symptom severity, and how different strains are introduced or persist over time in this region.

## Introduction

Human respiratory syncytial virus (RSV) is a leading cause of severe lower respiratory tract infections in young children. RSV causes an estimated 3.2 million annual hospitalizations and 60 000 deaths globally, primarily in children younger than 1 year old [1]. In general, the infection in the first year of life is the most severe [2]. RSV also contributes to severe acute respiratory disease in the elderly, comparable to that of influenza [1, 3–5]. A vaccine to protect infants and the elderly from RSV is a global health priority, and multiple vaccine candidates and mAbs, protective against both antigenic subgroups, are in different stages of development [6, 7]. Palivizumab is currently the only preventive mAb that protects against severe RSV acute respiratory infection [8]. In preparation for future vaccine roll-outs, there have been expanded efforts to understand the evolution and genetic diversity of the virus, including whole-genome sequencing [9–11].

RSV is an o*rthopneumovirus* in the newly defined family *Pneumoviridae* [12]. The viral genome consists of a negative ssRNA (~15 kb) strand that encodes 11 proteins. Two of the proteins are glycosylated, enabling fusion and attachment (F and G proteins, respectively) [7]. Two antigenic subgroups, RSVA and RSVB, are defined based on mAb reactivity to the glycoproteins [13]. Both antigenic subgroups co-circulate worldwide, causing seasonal epidemics, although RSVA is thought to be more prevalent and cause more severe illness than RSVB [11]. However, a higher prevalence of RSVB than of RSVA has been reported in several studies [14–16], and further understanding of the prevalence and immunological interactions between the antigenic subgroups is needed. Models of alternating epidemics have been proposed, driven by waning herd immunity [15, 17].

The molecular epidemiology of the virus has been characterized in multiple regions [10, 18–20], but there is a paucity of genetic sequence data for RSV, at least relative to influenza, and it remains unknown where new viral lineages originate and what drives their emergence. New RSV strains periodically emerge and become globally dominant, and 10 genotypes for RSVA and 13 RSVB genotypes have been identified to date [21, 22]. In the 1990s, a new RSVB genotype (BA) emerged with a duplication in the G gene and quickly disseminated globally [23, 24]. During the 2010–2011 winter season, a duplication in the G gene was detected among RSVA GA2 viruses, giving rise to the ON1 sub-genotype that also spread worldwide and became dominant in many countries [24–32]. The clinical impact of different genotypes has not been fully elucidated, as some studies report differences in disease severity [33–38], while others have seen similar clinical illness [36–38].

Intensive surveillance of RSV in infants in a paediatric cohort in Amman, Jordan, has revealed a high burden of RSV in hospitalized children [39–41]. To further understand the molecular evolution and transmission of RSV in this population, we obtained whole-genome sequences from 85 RSV samples collected during 2010–2013. We found that RSV epidemics in Jordan comprised multiple independent viral introductions of RSVA and RSVB, although one subgroup strongly dominated each year.

## Methods

### Sample and clinical data collection, and RSV diagnosis

All procedures were carried out in accordance with the ethical standards of the Jordanian Ministry of Health, University of Jordan and Vanderbilt University institutional review boards. After consent, trained local research staff obtained nasal and throat swabs. Standardized questionnaires were used to record demographic, clinical and socio-economic data. Parents were queried in Arabic, and bilingual research staff transcribed the information into an English-language case report form at the time of the interview. Demographic data, including age, sex, race and ethnicity, were recorded at the time of enrolment. Viral surveillance with clinical and demographic data in children<2 years old admitted with respiratory symptoms and/or fever was conducted at the Al-Bashir Government Hospital, Amman, Jordan from March 16 2010 to March 31 2013 [40, 41]. Nasal and throat swabs were collected and combined in transport medium (M4RT; Remel), aliquoted into MagMAX lysis/binding solution concentrate (Life Technologies), snap frozen, and stored at −80 °C. Original and lysis buffer aliquots were shipped on dry ice and were tested by reverse transcriptase (RT)-PCR for 11 respiratory viruses [RSV, human metapneumovirus (HMPV), human rhinovirus (HRV), influenza (flu) A, B and C, parainfluenza (PIV) virus 1, 2 and 3, adenovirus (ADV), and Middle East respiratory syndrome coronavirus (MERS-CoV)] [40].

From the 2010–2013 study period, a total of 3168 children's respiratory samples were processed for viral diagnostics, where>80 % tested positive for at least one virus, with RSV being the most common virus detected (44 %). Out of 1397 RSV-positive samples, 93 were randomly selected from all the 3 years with quantitative RT-PCR *C*_t_ value <29 and were processed for whole-genome next-generation sequencing of RSV. Out of the 93 samples, 7 samples were taken from study subjects who had died due to RSV disease.

### Viral RNA extraction, whole-genome sequencing, assembly and annotation

Extraction of the viral RNA was performed using 140 µl nasal wash sample in viral transport medium using the viral RNA mini kit (Qiagen). Four forward reverse transcription (RT) primers were designed and four sets of PCR primers were manually picked from primers designed across a consensus of complete RSV genome sequences as described before [11]. The four forward RT primers were diluted to 2 µM in water and pooled in equal volumes. cDNA was generated from 4 µl undiluted RNA, using the pooled forward primers and SuperScript III reverse transcriptase (Thermo Fisher Scientific). Four independent PCR reactions were performed on 2 µl cDNA template using either AccuPrime *Taq* DNA polymerase (Thermo Fisher Scientific) or Phusion high fidelity DNA polymerase (New England Biolabs) to generate four overlapping ~4 kb amplicons across the genome. Amplicons were verified with 1 % agarose gels, and excess primers and dNTPs were removed by treatment with exonuclease I (New England Biolabs) and shrimp alkaline phosphatase (Affymetrix) for 37 °C for 60 min, followed by incubation at 72 °C for 15 min. Amplicons were quantitated using a SYBR Green dsDNA detection assay (SYBR Green I nucleic acid gel stain; Thermo Fisher Scientific), and all four amplicons per genome were pooled in equal concentrations.

Samples were sequenced using both MiSeq (Illumina) and Ion Torrent PGM (Thermo Fisher Scientific) to overcome platform-specific errors. For the Illumina sequencing, libraries were prepared using the Nextera DNA sample preparation kit (Illumina) with half reaction volumes for MiSeq. Briefly, 25 ng pooled DNA amplicons were tagmented at 55 °C for 5 min. Tagmented DNA was cleaned with the ZR-96 DNA clean and concentrator kit (Zymo Research) and eluted in 25 µl resuspension buffer. Illumina sequencing adapters and barcodes were added to tagmented DNA via PCR amplification, where 20 µl tagmented DNA was combined with 7.5 µl Nextera PCR master mix, 2.5 µl Nextera PCR primer cocktail and 2.5 µl each index primer (Integrated DNA Technologies) for a total volume of 35 µl per reaction. Thermocycling was performed with 5 cycles of PCR, as per the Nextera DNA sample preparation kit protocol (3 min at 72 °C, denaturation for 10 s at 98 °C, annealing for 30 s at 63 °C and extension for 3 min at 72 °C) to create a dual-indexed library for each sample. After PCR amplification, 10 µl each library was pooled into a 1.5 ml tube, and the pool was cleaned twice with Ampure XP reagent (Beckman Coulter) to remove all leftover primers and small DNA fragments. The first cleaning used a 1.2× volume of the Ampure reagent, while the second cleaning used a 0.6× volume of the Ampure reagent. The cleaned pool was sequenced on the Illumina MiSeq v2 instrument (Illumina) with 300 bp paired-end reads.

In addition to Illumina sequencing, for Ion Torrent PGM (Thermo Fisher Scientific), 100 ng pooled DNA amplicons were sheared for 7 min and Ion-Torrent-compatible barcoded adapters were ligated to the sheared DNA using an Ion Xpress Plus fragment library kit (Thermo Fisher Scientific) to create 400 bp libraries. Libraries were pooled in equal volumes and cleaned with Ampure XP reagent (Beckman Coulter). Quantitative PCR was performed on the pooled, barcoded libraries to assess the quality of the pool and to determine the template dilution factor for emulsion PCR. The pool was diluted appropriately and amplified on Ion Sphere particles (ISPs) during emulsion PCR on an Ion One Touch 2 instrument (Thermo Fisher Scientific). The emulsion was broken, and the pool was cleaned and enriched for template-positive ISPs on an Ion One Touch ES instrument (Thermo Fisher Scientific). Sequencing was performed on the Ion Torrent PGM using 318v2 chips (Thermo Fisher Scientific).

### RSV genome assembly and annotation

Sequence reads were sorted by barcode, trimmed and *de novo* assembled using CLC Bio’s *clc assembler* program, formerly known as *clc novo assembly* (http://resources.qiagenbioinformatics.com/manuals/clcgenomicsworkbench/852/index.php?manual=De_novo_assembly.html), and the resulting contigs were searched against custom, full-length RSV nucleotide databases to find the closest reference sequence. All sequence reads were then mapped to the selected reference RSV sequence using CLC Bio’s *clc_mapper_legacy,* formerly called as *clc_ref_assemble_long* program (http://resources.qiagenbioinformatics.com/manuals/clcassemblycell/current/index.php?manual=Options_clc_mapper_legacy.html). At loci where both Ion Torrent and Illumina sequence data agreed on a variation (compared with the reference sequence), the reference sequence was updated to reflect the difference. A final mapping of all next-generation sequences to the updated reference sequences was performed with CLC Bio’s *clc_mapper_legacy* program. Curated assemblies were validated and annotated with the viral annotation software called Viral Genome ORF Reader, VIGOR 3.0 [42], before submission to GenBank. VIGOR was used to predict genes, perform alignments, ensure the fidelity of ORFs, correlate nucleotide polymorphisms with amino acid changes and detect any potential sequencing errors. The annotation was subjected to manual inspection and quality control before submission. All 85 sequences generated as part of this study were submitted to GenBank as part of the BioProject ID PRJNA262901 with accession number KX655615–KX655701 (KX655701, KX655688 and KX655625 are partial sequences).

### Dataset assembly and phylogenetic analyses

In order to enrich the datasets of 58 RSVA and 27 RSVB Jordanian full-genome sequences, we retrieved all RSV whole-genome sequences from GenBank on July 21 2016, resulting in datasets of 771 and 331 sequences for RSVA and RSVB, respectively. The data were aligned using mafft v7.310 [43] and subsequently manually edited to accommodate the ORFs of all genes and the G gene duplication. To identify recombinant strains that would complicate the phylogenetic inferences, we analysed the datasets using the Recombination Detection Program Beta v4.94 [44]. This resulted in the exclusion of one recombinant RSVA sequence (JX015495) and four recombinant RSVB sequences (KJ939932, KJ672473, KJ627251, KJ939933) from locations other than Jordan. Maximum-likelihood trees were inferred using RAxML v7.2.6 [45] incorporating a general time reversible (GTR) model of nucleotide substitution with a gamma-distributed (Γ) rate variation among sites. To assess the robustness of each node, a 500 replicates bootstrap resampling process was performed. We also investigated the temporal signal of the datasets using TempEst [46]. A few sequences showing incongruent temporal patterns were excluded. Phylogenetic relationships were inferred for each of the data sets separately (669 RSVA sequences and 327 RSVB sequences) with a Bayesian phylogenetic approach using Markov Chain Monte Carlo available via the beast v1.8.4 package [47] and the high-performance computational capabilities of the Biowulf Linux cluster at the NIH, Bethesda, MD, USA (https://hpc.nih.gov/systems/). We used an uncorrelated relaxed molecular clock with branch rates drawn form a lognormal distribution to account for evolutionary rate variation among lineages, with a Skygrid demographic prior [48], and a GTR model of nucleotide substitution with gamma-distributed rate variation among sites. For viruses with only the year of viral collection available, the lack of tip date precision was accommodated by sampling uniformly across a 1 year window from January 1st to December 31st. The Markov chain Monte Carlo (MCMC) chain was run separately at least three times for each of the data sets and for at least 200 million iterations with sub-sampling every 20 000 iterations, using the beagle [49] library to improve computational performance. All parameters reached convergence, as assessed visually using Tracer v.1.6.0, with statistical uncertainty reflected in values of the 95 % highest posterior density (HPD). At least 10 % of the chain was removed as burn-in. Maximum clade credibility (MCC) trees were summarized using TreeAnnotator v1.8.4 and the trees were visualized in FigTree v1.4.3. The samples were collected from different neighbourhood zones in Amman, Jordan (11 and 9 zones for the antigenic subgroup A and B, respectively), which were aggregated into four spatial regions within Amman (North, Central, North-East and West regions) for our spatial analysis. To study the proportion of RSVA and RSVB circulating in the Amman population and globally, we merged the datasets of both RSV antigenic subgroups and estimated the proportion of RSVA and RSVB viruses, and their respective genotypes, circulating in 15 countries over time, using the R program [50].

### Clinical data

We collected information on the patient age and bronchopneumonia status for the Amman paediatric cohort during 2010–2013. As we had collected this information, we investigated whether infection with a specific RSV antigenic subgroup was significantly associated with patient age or disease progression to bronchopneumonia. For this purpose, we used two statistical tests, namely the *t*-test (patient age as a continuous variable) and the chi-square test (bronchopneumonia as a binary outcome). Additionally, we also investigated the extent of RSV phylogenetic structure for bronchopneumonia (discrete trait) using the association index (AI). This metric quantifies the degree to which samples of patients with bronchopneumonia tend to cluster together relative to the expectation for randomized trait assignments. AI values close to 0 reflect strong phylogeny-trait correlation, whereas AI values close to 1 reflect the absence of phylogenetic structure for the trait [51]. For this purpose, we reconstructed the bronchopneumonia status at the ancestral nodes using the discrete diffusion models implemented in beast [52].

## Results

### Genetic diversity of RSV in a paediatric cohort in Amman, Jordan

**Fig. 1.**
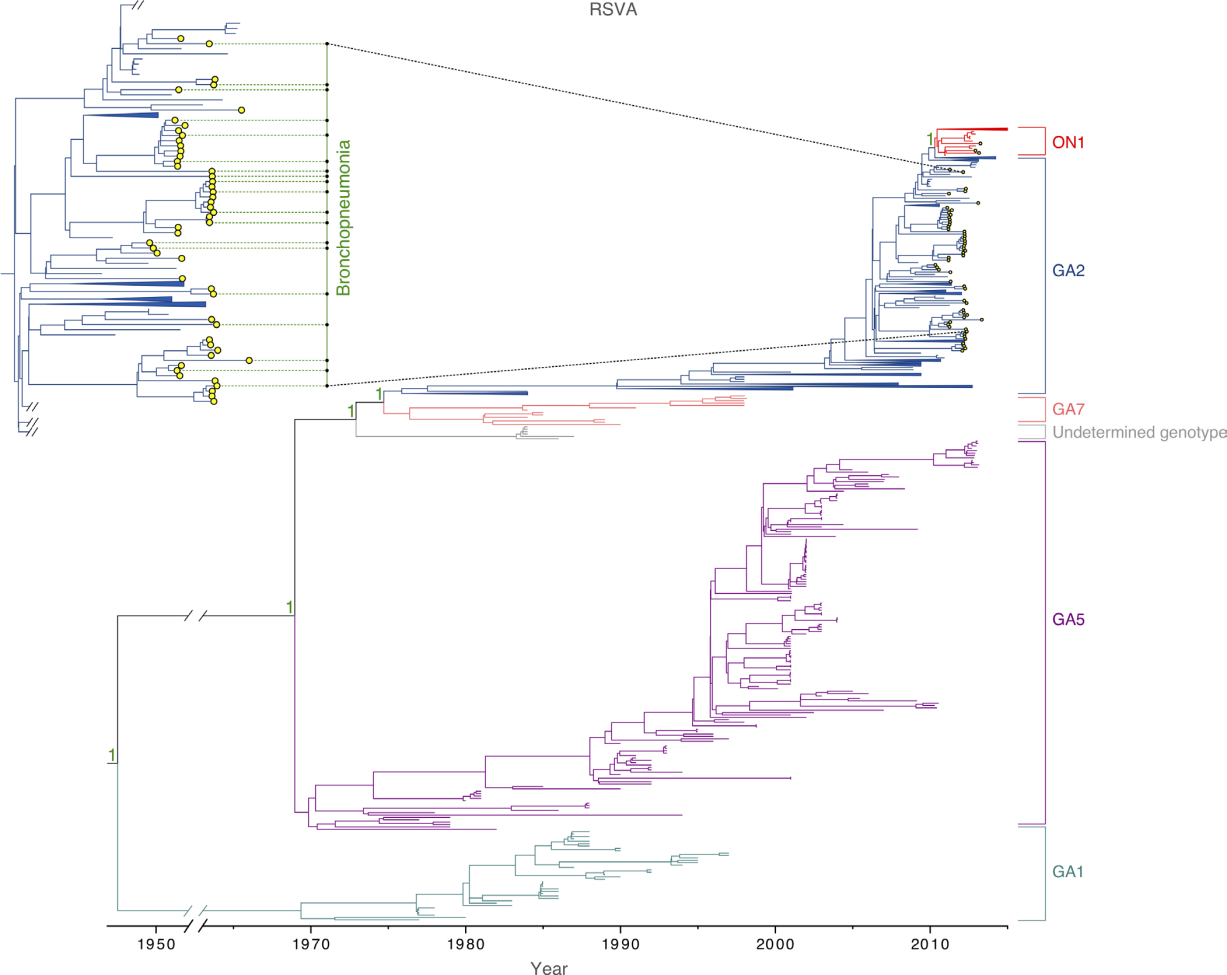
Global RSVA phylogeny. MCC tree inferred for 669 viruses sampled globally, coloured by genotype. The yellow-filled circles indicate 58 samples collected from a paediatric cohort in Amman, Jordan, which cluster within the GA2 genotype and ON1 sub-genotype. A few tips from countries other than Jordan are collapsed for visual clarity. Green dotted lines indicate samples from patients with bronchopneumonia. Posterior probabilities are indicated as support for nodes defining a genotype. A more detailed tree of RSVA samples collected in Amman is provided in Fig. 4.

**Fig. 2.**
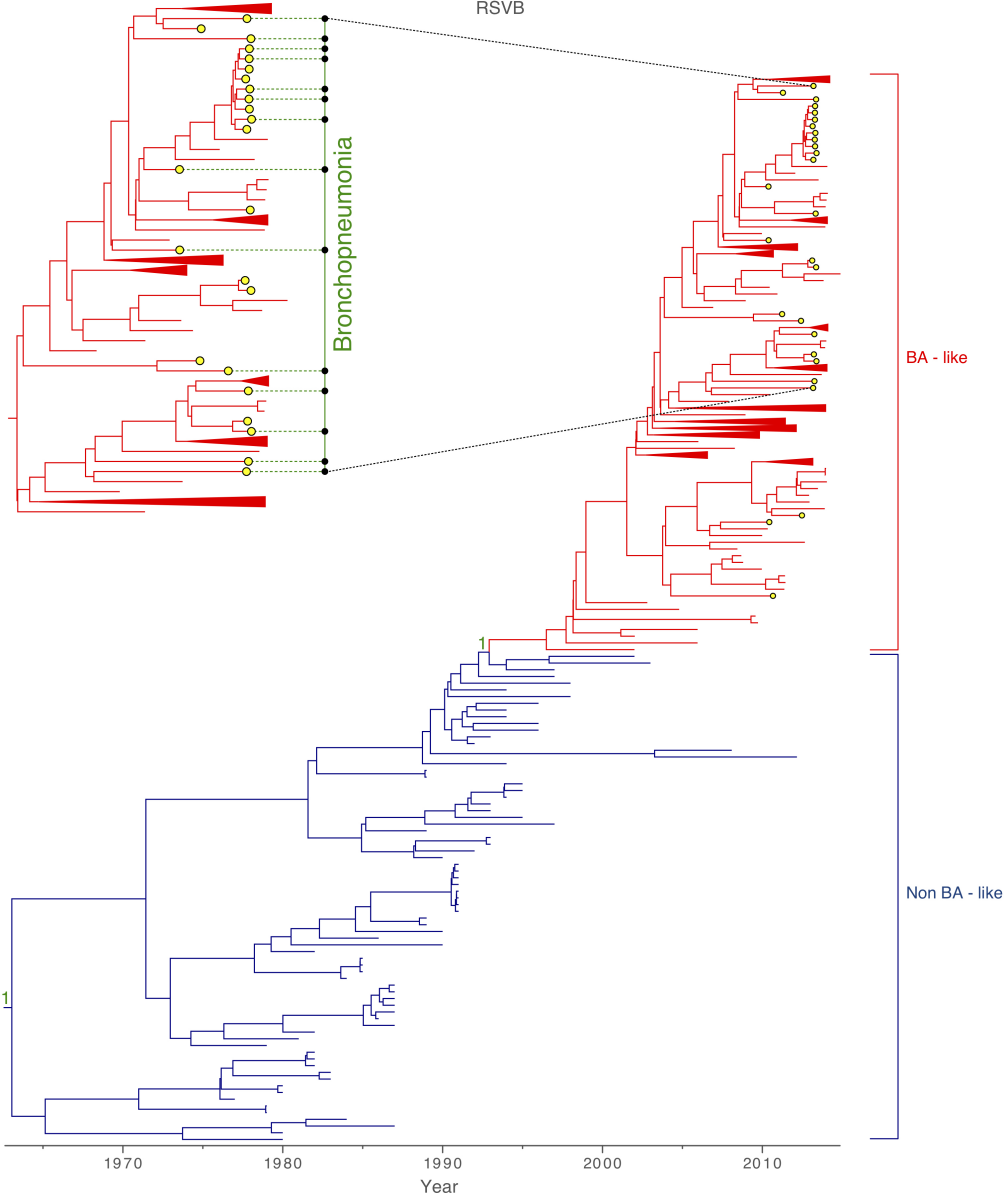
Global RSVB phylogeny. MCC tree inferred for 327 viruses sampled globally, coloured by genotype. The yellow-filled circles indicate 27 samples collected from a paediatric cohort in Amman, Jordan, which cluster within the GB1 sub-genotype positioned within the BA-like clade. A few tips from countries other than Jordan are collapsed for visual clarity. Green dotted lines indicate samples from patients with bronchopneumonia. Posterior probabilities are indicated as support for nodes defining a genotype. A more detailed tree of RSVB samples collected in Amman is provided in Fig. 4.

**Fig. 3.**
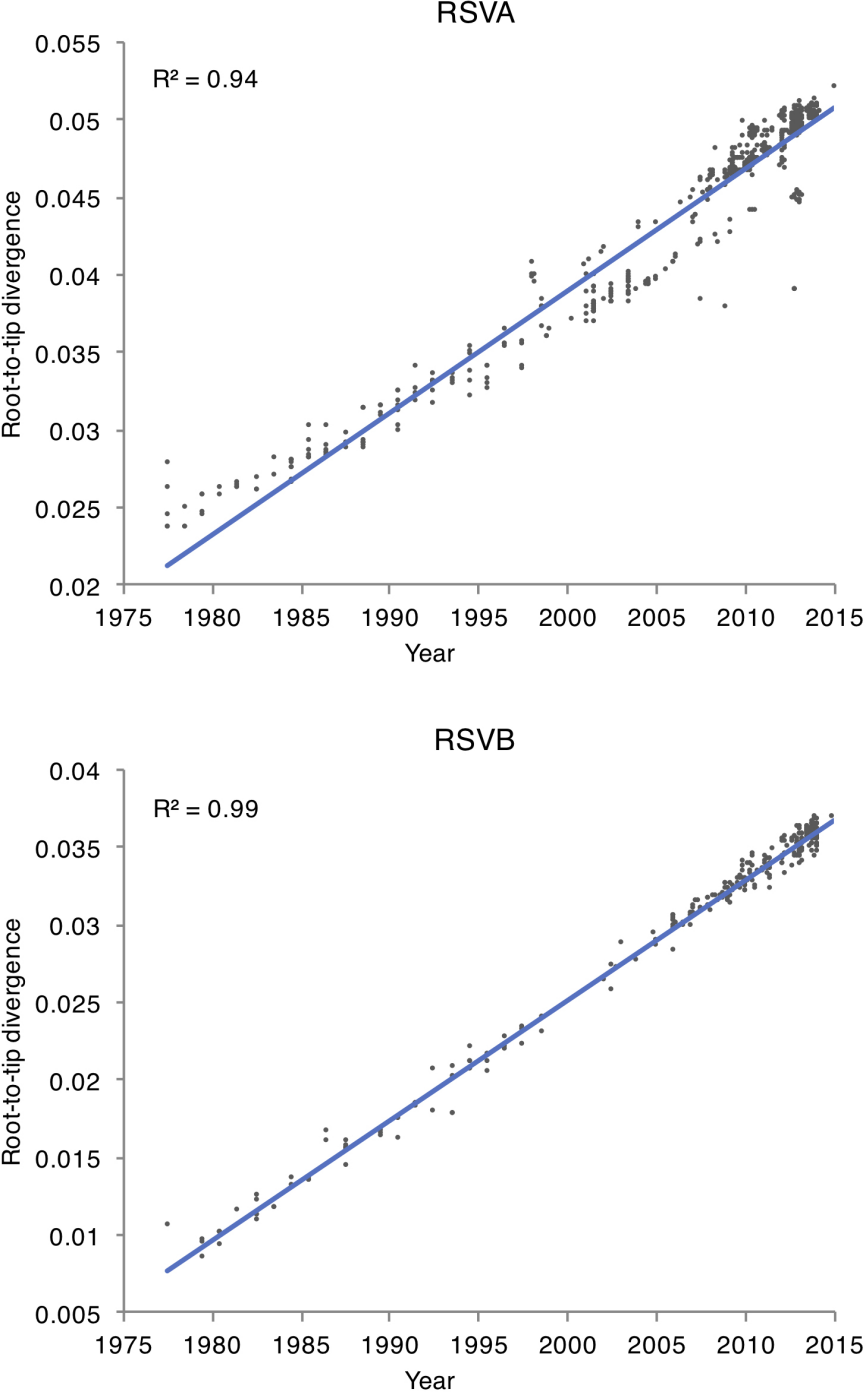
Root-to-tip divergence. A plot of sampling time versus genetic distance, inferred from trees of RSVA and RSVB inferred using maximum-likelihood methods. Both RSVA and RSVB datasets display a strong temporal signal for a molecular clock, as shown by the high *R* ^2^.

Among the 93 samples for which sequencing was attempted, 85 complete viral genomes were successfully sequenced from the Amman paediatric cohort during 2010–2013, 58 were identified as RSVA and 27 as RSVB. The mean age of patients with RSVA was 2.5 years (9.3 months – 5.4 years), and 1.8 years (8.3 months – 4.8 years) for RSVB. Most of the RSVA isolates collected in Amman belonged to the dominant GA2 genotype (55/58). Three samples, collected during October 2012 – February 2013, were positioned within the newly emerged ON1 sub-genotype, which is characterized by a 72 nucleotide duplication at the G gene [25] (Fig. 1). All RSVB samples were sub-genotyped as GB1 and positioned within the BA-like clade, which is defined by a duplication of 60 nucleotides in the G gene (Fig. 2) [23, 53]. A plot of genetic distance against time indicates that the evolution of RSVA and RSVB is strongly clock-like for both viruses, with correlation coefficients of 0.94 and 0.99, respectively (Fig. 3). RSVA and RSVB were found to be evolving at similar rates [RSVA 7.31×10^−4^ (95 % HPD 6.78×10^−4^ – 7.88×10^−4^) substitutions/site/year; RSVB 7.08×10^−4^ (95 % HPD 6.58×10^−4^ – 7.59×10^−4^) substitutions/site/year) (Table 1), estimates that are consistent with previous studies [11, 19]. However, the genetic diversity of RSVA traces back to a common ancestor that existed approximately 27 years earlier than RSVB (Table 1).

**Table 1.** Evolutionary rates and times of divergence for RSVA and RSVB Values in parentheses represent 95 % HPD intervals.

RSV	Evolutionary rate (substitutions/site/year)	tMRCA (date in decimal format)
RSVA	7.31×10^−4^ (6.78×10^−4^ – 7.88×10^−4^)	1937.54 (1917.58 – 1954.26)
RSVB	7.08×10^−4^ (6.58×10^−4^ – 7.59×10^−4^)	1964.79 (1961.58 – 1967.84)

### Multiple introductions of RSV into the Amman cohort during each epidemic

**Fig. 4.**
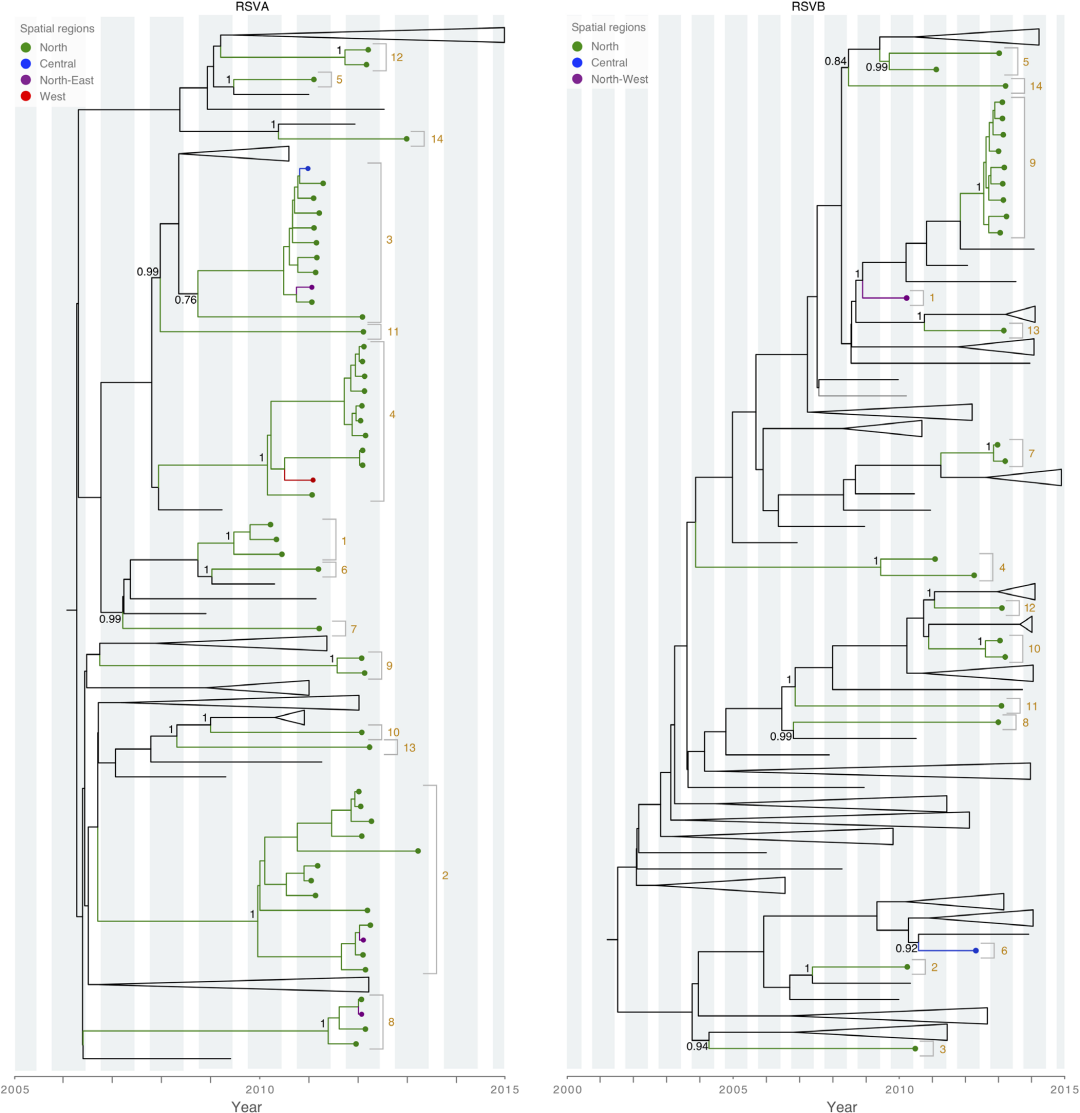
Spatial patterns of RSV introductions in Amman, Jordan. Truncated MCC trees (RSVA, left; RSVB, right) with tip labels coloured by spatial region in Amman, Jordan. Tips from countries other than Jordan are collapsed for visual clarity. Discrete viral introductions are numbered, similarly to Tables 2 and 3, and shaded orange. Node support is indicated for posterior probabilities >0.75. Vertical grey bars indicate RSV epidemic seasons in Jordan, according to Halasa *et al*. [40].

Our study period included four winter epidemic seasons, defined as September to June: 2009–2010, 2010–2011, 2011–2012 and 2012–2013. We found that each annual RSV epidemic in Amman was not a point-source outbreak, but instead seeded by multiple independent viral introductions, including both RSVA and RSVB lineages, into the Amman cohort population. The low availability of RSV sequence data from many countries makes it difficult to infer the precise number of viral introduction, but we estimated, conservatively, the number of introductions in each year (Fig. 4): five introductions during the 2009–2010 epidemic, eight during the 2010–2011 epidemic, seven during the 2011–2012 epidemic, and nine during the 2012–2013 epidemic. Despite the higher availability of RSVA sequences, the same number of total introductions of RSVA and RSVB were identified during the 4 years of study (*n*=14). Three RSVA (ON1) were identified during the 2012–2013 epidemic, representing at least one additional introduction since they belong to an entirely different genotype, but are not well supported on the tree. Viruses from multiple epidemic seasons sometimes clustered together, possibly representing persistence of RSV in Amman over multiple years. Similarly, the time to the most recent common ancestor (tMRCA) values were estimated to exceed 1 year for several viral introductions, based on the time-scaled MCC trees (Tables 2 and 3). However, the low availability of background RSV sequences from other locations means that we cannot exclude the possibility that genetically close viruses were re-imported each year from an unsampled location, rather than persisting locally.

**Table 2.** Highly supported RSVA introductions in Amman, Jordan

Introduction	No. of samples	Posterior probability	Spatial region	tMRCA (date in decimal format)*	Viral persistence	Genotype
1	3	1	North	2009.56 (2009.18 – 2009.91)	3 months	GA2
2	13	1	North, North-East	2009.56 (2009.02 – 2010.19)	2 years and 2 months	GA2
3	11	0.76	North, Central, North-East	2008.65 (2008.07 – 2009.24)	1 year and 2 months	GA2
4	11	1	North, West	2009.91 (2009.38 – 2010.39)	1 year and 1 month	GA2
5	1	1	North	–	–	GA2
6	1		North	–	–	GA2
7	1	0.99	North	–	–	GA2
8	4	1	North, North-East	2011.21 (2010.72 – 2011.63)	2 months	GA2
9	2	1	North	2011.80 (2011.48 – 2012.04)	1 month	GA2
10	1	1	North	–	–	GA2
11	1	0.99	North	–	–	GA2
12	2	1	North	2011.74 (2011.33 – 2012.07)	13 days	GA2
13	1	1	North	–	–	GA2
14	1	1	North	–	–	GA2

*Values in parentheses represent 95 % HPD intervals

**Table 3.** Highly supported RSVB introductions in Amman, Jordan

Introduction	No. of samples	Posterior probability	Spatial region	tMRCA (date in decimal format)*	Viral persistence	Genotype
1	1	1	North-West	–	–	GB1
2	1	1	North	–	–	GB1
3	1	0.94	North	–	–	GB1
4	2	1	North	2009.31 (2008.34 – 2010.22)	1 year and 3 months	GB1
5	2	0.99	North	2009.86 (2009.26 – 2010.36)	1 year and 11 months	GB1
6	1	0.92	Central	–	–	GB1
7	2	1	North	2012.86 (2012.71 – 2012.96)	3 months	GB1
8	1	0.99	North	–	–	GB1
9	9	1	North	2012.46 (2012.22 – 2012.67)	3 months	GB1
10	2	1	North	2012.63 (2012.33 – 2012.89)	2 months	GB1
11	1	1	North	–	–	GB1
12	1	1	North	–	–	GB1
13	1	1	North	–	–	GB1
14	1	0.84	North	–	–	GB1

*Values in parentheses represent 95 % HPD intervals.

### Spatial patterns of RSVA and RSVB in Amman

To study the spatial dissemination of RSV within the Amman cohort population, four regions were defined among the city’s neighbourhoods from which viruses were successfully sequenced (Fig. 4). Four of the fourteen RSVA introductions were identified in multiple regions, consistent with spatial spread within the city. One RSVA introduction was identified in three of the four spatial regions defined within Amman. In contrast, although a large number of introductions of RSVB into Amman were identified (*n*=14), each introduction was confined to a single city region, and there was no evidence of dissemination of RSVB between regions (Tables 2 and 3).

### Proportion of circulating RSV antigenic subgroups A and B

**Fig. 5.**
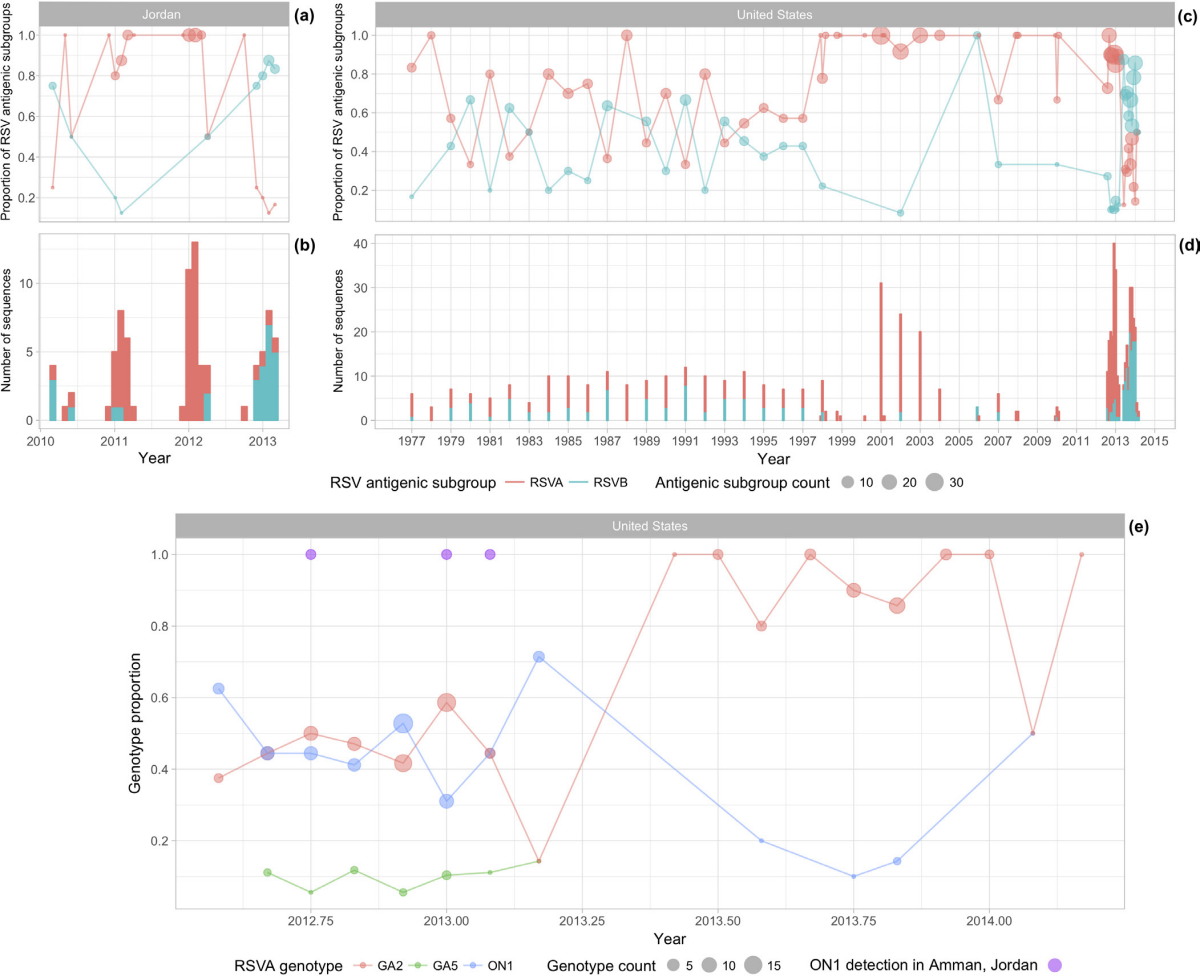
Circulation of RSV in Jordan and the USA. The proportion of all available full-genome RSV sequences aggregated by antigenic subgroup for Jordan collected from 2010 to 2013 (a), for the USA from 1977 to 2014 (c) and aggregated by genotype for the USA from 2012 to 2014 (e). Circle sizes represent the number of sequences from each antigenic subgroup (**a and **c) or genotype (e) contributing to the proportion. Total number of RSV sequences from Jordan collected from 2010 to 2013 (b) and from the USA from 1977 to 2014 (d). Purple circles represent dates when genotype ON1 was identified in Amman, Jordan.

We investigated the frequency and distribution of RSV cases during the 2010–2013 period (Fig. S3) and observed limited variation across seasons. RSVA and RSVB co-circulated in Amman, but one antigenic subgroup dominated over the other during an epidemic. RSVA was dominant during the 2010/2011 and 2011/2012 winter epidemics, and RSVB was dominant during the 2009/2010 and 2012/2013 winter epidemics (Fig. 5a, b). Alternating dominance between RSVA and RSVB was also observed in the USA, the only country with a sufficiently long time series of genetic data to examine genotype interactions (Fig. 5c, d). The low availability of ON1 sequences in Jordan (*n*=3) prevented a similar examination of interactions between the RSVA ON1 and GA2 genotypes within Jordan. However, Fig. 5(e) shows that the cluster of ON1 viruses in Jordan during the 2012/2013 winter epidemic coincided with the emergence of ON1 in the USA.

### Association of viral genetics and clinical outcomes

RSVA-infected children tended to be older than RSVB-infected children (30 months versus 22.4 months, respectively) (two-tailed *t*-test *P* value=0.02) in this cohort. Approximately 32.8 % of RSVA-infected and 51.9 % of RSVB-infected patients developed bronchopneumonia (Figs 1 and 2), but this difference was not found to be significant (chi-square *P* value=0.06), given the small sample size. We observed little phylogenetic clustering by whether a child developed bronchopneumonia, with AI values very close to 1 for both antigenic subgroups: RSVA, AI=0.98 (95 % HPD interval 0.78–1.21); RSVB, AI=1.16 (95 % HPD interval 0.86–1.50).

## Discussion

RSV imposes a high burden of respiratory disease on infants and children in Amman, Jordan [40]. By sequencing the complete genomes of 85 RSVA and RSVB samples from children in Amman, the largest whole-genome collection of RSVs from any country in the Middle East, this study provides insights into the genetic diversity of RSVA and RSVB in an under-studied region. We found that RSV epidemics in Amman comprised multiple co-circulating strains of both RSVA and RSVB, as observed in other regions [54], although one subgroup tends to strongly dominate (RSVA dominated seasons 2010/2011 and 2011/2012, and RSVB dominated seasons 2009/2010 and 2012/2013). The severity of clinical outcomes is difficult to predict from the genetic sequence of the virus alone, at least with the sample size used in this study, although the higher frequency of bronchopneumonia associated with RSVB (51.9 %) compared with RSVA (32.8 %) merits further study. The vast majority of RSV samples in Amman belonged to the most predominant RSVA and RSVB genotypes (GA2 and BA-like, respectively), with only three RSVA identified from the recently emerged ON1 sub-genotype. More intensive sequencing may be needed to detect lower frequency variants.

Our study benefitted from using whole-genome sequences, as the partial G gene may produce misleading results [53] and transmission patterns may be difficult to infer using shorter coding regions [19]. However, at this time there is far more background RSV sequence data available for the G gene for other countries, compared to whole-genome data, which limited our ability to differentiate independent viral introductions into Amman during an epidemic and infer their spatial origins. Compared to influenza A viruses, for which the global spatial ecology has been extensively characterized [55, 56], the paucity of RSV sequence data from many regions undermines efforts to understand how the virus moves spatially around the world between local epidemics. It is clear that RSV is continually being re-introduced into Amman, similar to the viral dynamics observed in other longitudinal studies of RSV, such as in Kenya [57]. However, it remains unclear whether global RSV evolution follows a metapopulation model or a source-sink model, and the extent to which viruses persist in a single location. Additionally, the limited number of RSV samples from Jordan prevented more sophisticated phylogeographical modelling that could unveil the dynamics within the city. In the future, it would be particularly interesting to compare the spatial dynamics of RSVA and RSVB. Our analysis revealed more instances of RSVA transmission across multiple regions of the city, but these inferences were based on very low sample sizes, particularly for RSVB, and require further study with larger data sets.

The ongoing development of candidate RSV vaccines underscores the need for further understanding of human immune responses, and the extent of cross-immunity between RSV strains of varying genetic distances. The potential interactions between RSVA and RSVB observed in Jordan and the USA, with sequential alternation in the transmission of antigenic subgroups and genotypes (Fig. S5), may result in subgroup- or genotype-specific immunity. These dynamics demonstrate that future vaccine compositions could benefit by including representative strains for both RSVA and RSVB, or even, representative strains for the different genotypes. Additionally, there is a need for further sequencing of RSV over sufficiently long time-series within discrete locations to characterize population dynamics and estimate competitive immunological interactions. The recent emergence of ON1 variants also provides an opportunity to examine competitive interactions between ON1 and previously dominant GA2 strains across multiple geographical locations, to better understand the strength and breadth of immune responses to different RSV strains. Going forward, long-term studies of RSV dynamics within defined populations will be essential to inform the design and potential impact of future RSV vaccines.

## Data bibliography

Trovão N. S. Accession numbers of the genetic sequences used in this study are listed in the Supplementary material, available with the online version of this article (2019).

## Supplementary Data

Supplementary material 1
