# Supplementary material for: Molecular characterization of respiratory syncytial viruses circulating in a paediatric cohort in Amman, Jordan
Source: Microb Genom. 2019 Sep 18;7(6):000292. doi: 10.1099/mgen.0.000292 (PMC8627666; doi:10.1099/mgen.0.000292)
Supplement: Supplementary material 1 [file mgen-7-0292-s001.pdf]

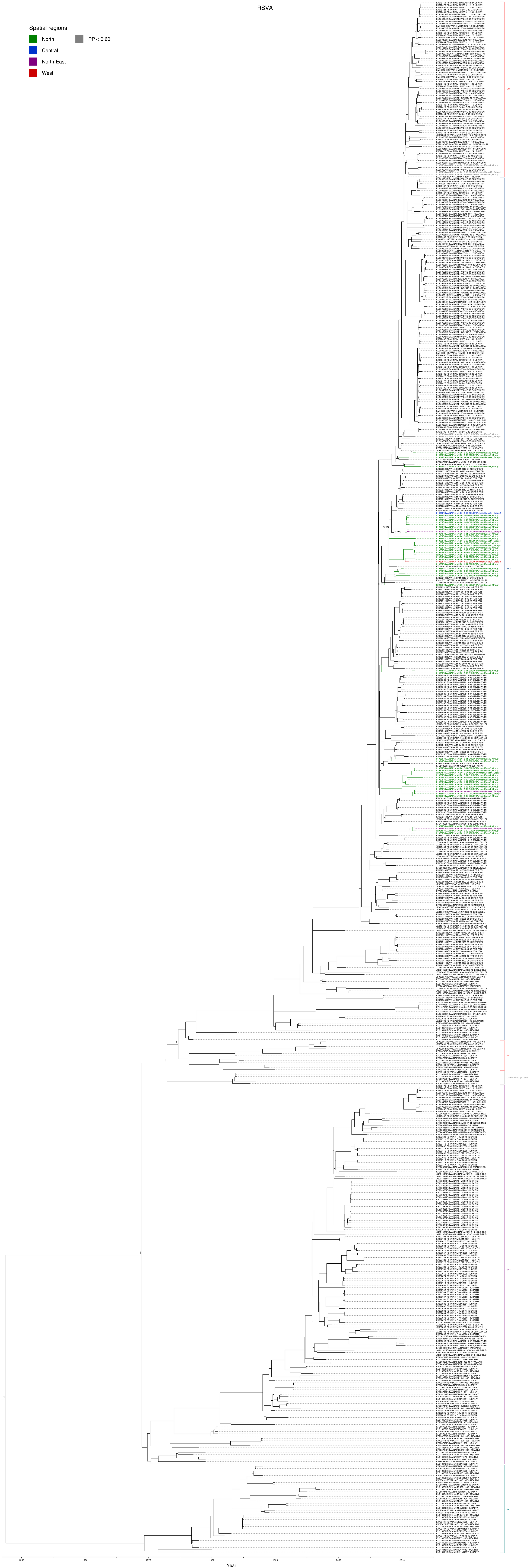

RSVA
Spatial regions
North
Central
North-East
West
PP < 0.60
1950
1960
1970
1980
1990
2000
2010
Year
GA1

Supplementary Figure 1. RSVA phylogeny indicates spatial regions. MCC tree with tip labels colored per spatial region in Amman, Jordan, and annotated with genotype and node support as posterior probability > 0.75.

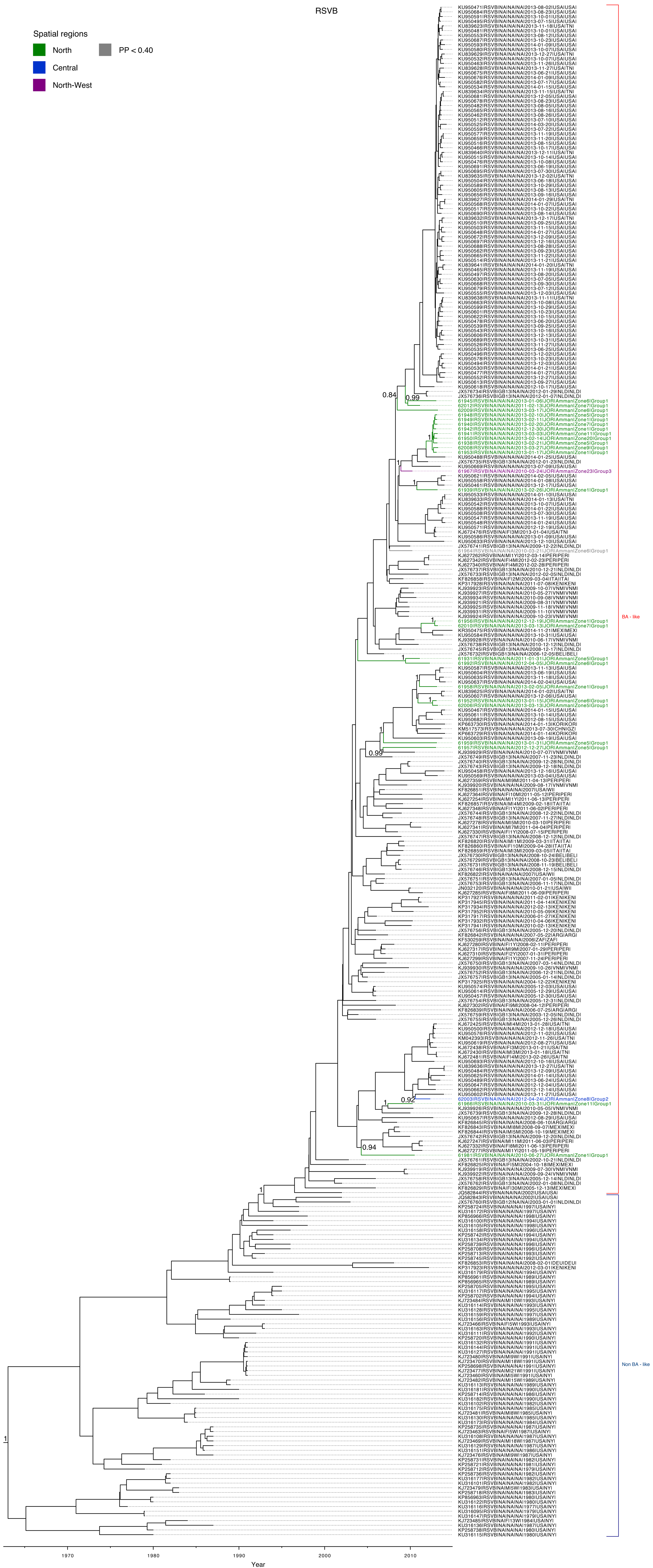
RSVB
Spatial regions
North
Central
North-West
PP < 0.40
BA - like
Non BA - like
1970
1980
1990
2000
2010
Year

Supplementary Figure 2. RSVB phylogeny indicates spatial regions. MCC tree with tip labels colored per spatial region in Amman, Jordan, and annotated with genotype and node support as posterior probability > 0.75.

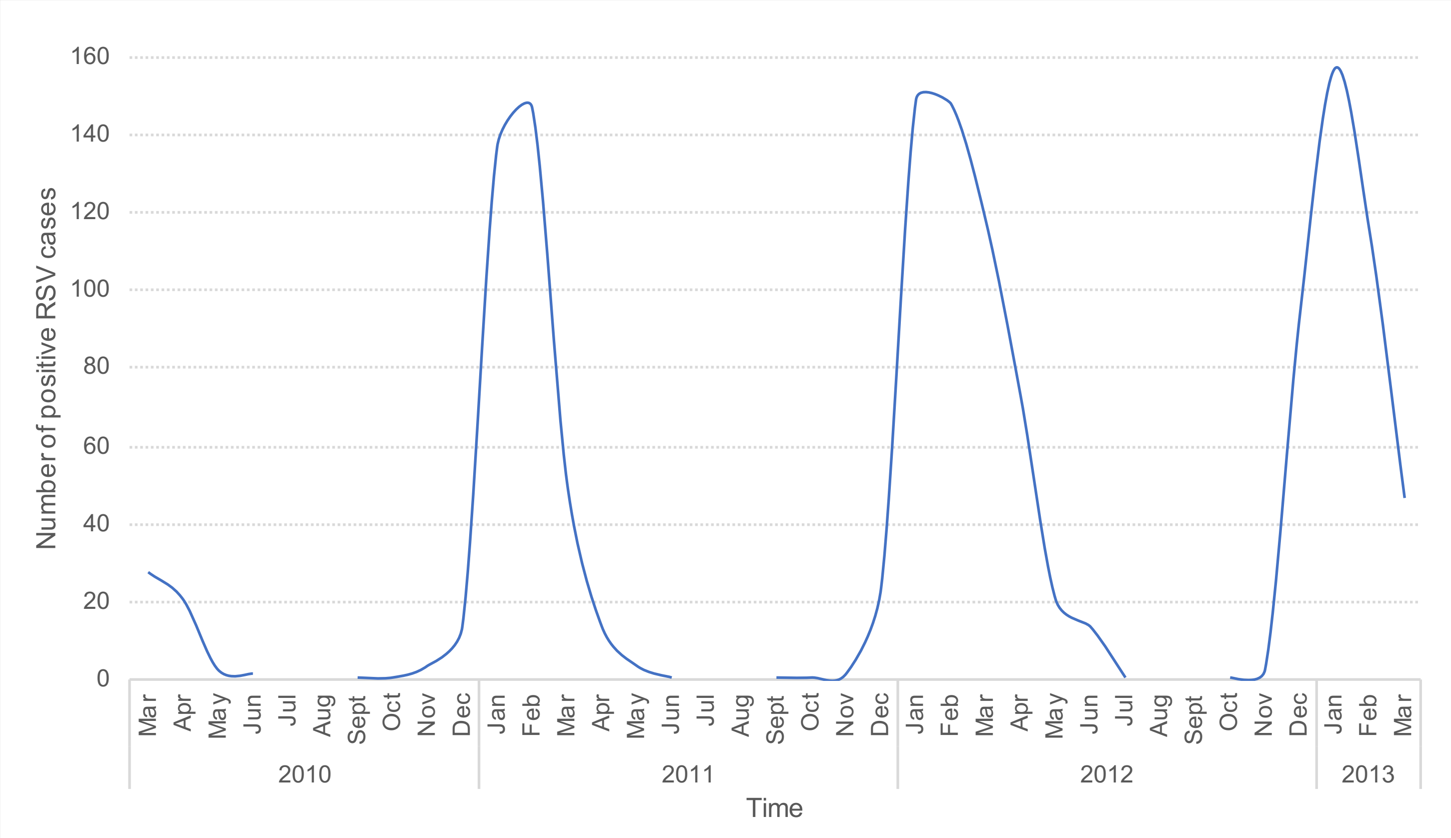

Number of positive RSV cases
160
140
120
100
80
60
40
20
0
Mar
Apr
May
Jun
Jul
Aug
Sept
Oct
Nov
Dec
Jan
Feb
Mar
Apr
May
Jun
Jul
Aug
Sept
Oct
Nov
Dec
Jan
Feb
Mar
Apr
May
Jun
Jul
Aug
Sept
Oct
Nov
Dec
Jan
Feb
Mar
2010
2011
2012
2013
Time

Supplementary Figure 3. RSV cases in Amman, Jordan. The distribution of the total number of RSV cases detected over three years by month in Amman, Jordan.

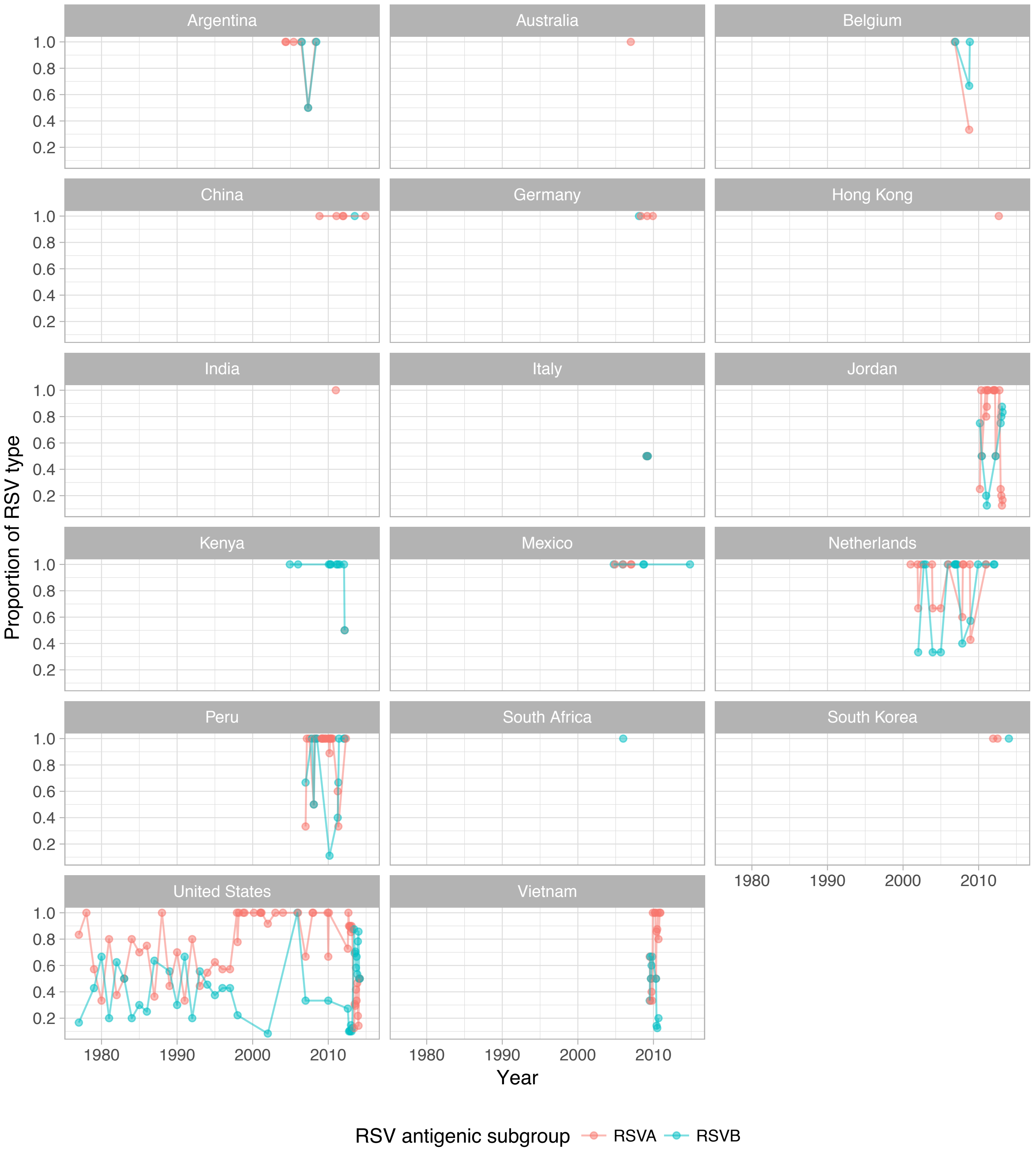

Argentina
Australia
Belgium
China
Germany
Hong Kong
India
Italy
Jordan
Kenya
Mexico
Netherlands
Peru
South Africa
South Korea
United States
Vietnam
1.0
0.8
0.6
0.4
0.2
1980
1990
2000
2010
Proportion of RSV type
Year
RSV antigenic subgroup
RSVA
RSVB

Supplementary Figure 4. Circulation of RSV worldwide. The proportion of all available full-genome RSV sequences from all sampled countries from 1977-2014.

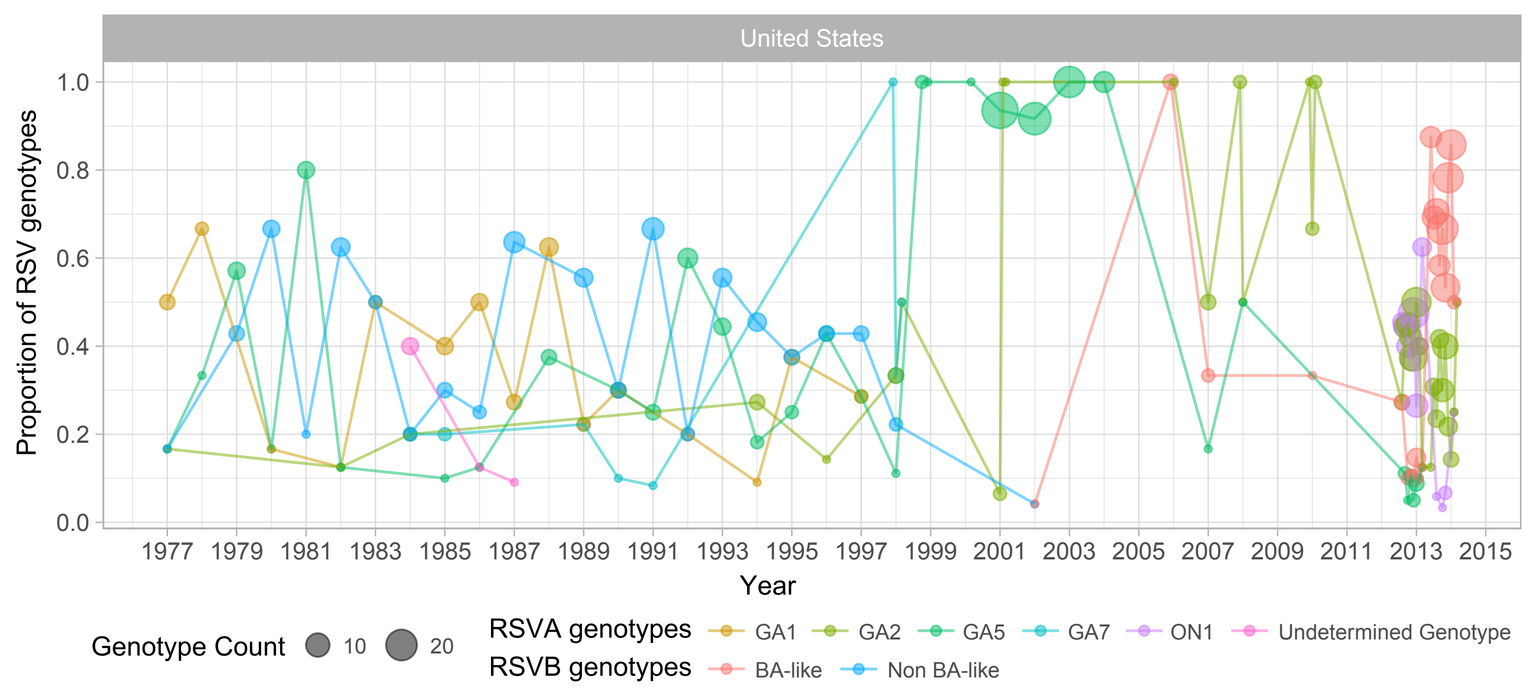
United States
Proportion of RSV genotypes
1.0
0.8
0.6
0.4
0.2
0.0
1977
1979
1981
1983
1985
1987
1989
1991
1993
1995
1997
1999
2001
2003
2005
2007
2009
2011
2013
2015
Year
Genotype Count
10
20
RSVA genotypes
GA1
GA2
GA5
GA7
ON1
Undetermined Genotype
RSVB genotypes
BA-like
Non BA-like

Supplementary Figure 5. Circulation of RSV genotypes in the United States. The proportion of all available full-genome RSV sequences aggregated by genotype for the United States from 1977-2014. Circle sizes represent the number of sequences from each genotype contributing for the proportion.

Supplementary File 1. RSV datasets. Sequences used in the study annotated with genotype. Jordanian sequences numbered according to introduction.

Taxa	Genotype	Introduction
KJ672451|RSVA|NA|M|5M|2012-12-27|USA|TN|	ON1	NA
KJ672470|RSVA|NA|M|3M|2012-12-18|USA|TN|	ON1	NA
KJ672466|RSVA|NA|M|5M|2012-12-05|USA|TN|	ON1	NA
KJ672424|RSVA|NA|F|1M|2012-12-27|USA|TN|	ON1	NA
KJ672461|RSVA|NA|M|4M|2012-12-31|USA|TN|	ON1	NA
KU950509|RSVA|NA|F|11W|2012-12-11|USA|USA|	ON1	NA
KU950680|RSVA|NA|M|9W|2012-11-29|USA|USA|	ON1	NA
KU950674|RSVA|NA|M|2W|2012-10-31|USA|USA|	ON1	NA
KU950660|RSVA|NA|F|9W|2012-08-31|USA|USA|	ON1	NA
KU950511|RSVA|NA|M|9W|2012-09-27|USA|USA|	ON1	NA
KM042383|RSVA|NA|M|5M|2012-12-31|USA|TN|	ON1	NA
KU950485|RSVA|NA|M|2W|2012-12-27|USA|USA|	ON1	NA
KU950572|RSVA|NA|F|2W|2012-09-28|USA|USA|	ON1	NA
KJ672452|RSVA|NA|M|1M|2013-01-07|USA|TN|	ON1	NA
KJ672480|RSVA|NA|M|2M|2013-01-23|USA|TN|	ON1	NA
KM042386|RSVA|NA|M|5M|2013-01-14|USA|TN|	ON1	NA
KU950480|RSVA|NA|F|2W|2012-12-10|USA|USA|	ON1	NA
KJ672464|RSVA|NA|M|3M|2012-12-13|USA|TN|	ON1	NA
KU950474|RSVA|NA|F|15W|2012-10-16|USA|USA|	ON1	NA
KU950567|RSVA|NA|F|2W|2012-12-03|USA|USA|	ON1	NA
KU950596|RSVA|NA|M|1W|2013-11-25|USA|USA|	ON1	NA
KU950627|RSVA|NA|M|9W|2013-10-25|USA|USA|	ON1	NA
KU839637|RSVA|NA|NA|NA|2014-02-03|USA|TN|	ON1	NA
KU950513|RSVA|NA|F|1W|2012-11-09|USA|USA|	ON1	NA
KU950492|RSVA|NA|M|2W|2012-10-03|USA|USA|	ON1	NA
KU950592|RSVA|NA|F|7W|2012-08-27|USA|USA|	ON1	NA
KU950692|RSVA|NA|F|3W|2012-09-26|USA|USA|	ON1	NA
KJ672441|RSVA|NA|F|3M|2013-03-21|USA|TN|	ON1	NA
KU950524|RSVA|NA|M|1W|2012-11-23|USA|USA|	ON1	NA
KM042389|RSVA|NA|M|1M|2012-12-13|USA|TN|	ON1	NA
KU950642|RSVA|NA|F|11W|2012-10-10|USA|USA|	ON1	NA
KJ672469|RSVA|NA|F|5M|2013-02-08|USA|TN|	ON1	NA
KM042388|RSVA|NA|F|5M|2013-01-11|USA|TN|	ON1	NA
KJ672475|RSVA|NA|M|3M|2013-01-28|USA|TN|	ON1	NA
KM042390|RSVA|NA|M|5M|2013-02-19|USA|TN|	ON1	NA
KJ672440|RSVA|NA|M|4M|2012-11-23|USA|TN|	ON1	NA

KJ672433|RSVA|NA|M|1M|2013-02-20|USA|TN|	ON1	NA
KU950673|RSVA|NA|M|1W|2012-09-12|USA|USA|	ON1	NA
KU950671|RSVA|NA|M|1W|2012-11-08|USA|USA|	ON1	NA
KU950653|RSVA|NA|F|8W|2012-12-06|USA|USA|	ON1	NA
KU950612|RSVA|NA|F|9W|2012-10-11|USA|USA|	ON1	NA
KU950696|RSVA|NA|M|12W|2012-12-19|USA|USA|	ON1	NA
KU950493|RSVA|NA|F|9W|2012-09-12|USA|USA|	ON1	NA
KU950626|RSVA|NA|F|9W|2013-01-28|USA|USA|	ON1	NA
KJ672468|RSVA|NA|M|1M|2012-11-19|USA|TN|	ON1	NA
KJ672429|RSVA|NA|F|2M|2013-02-12|USA|TN|	ON1	NA
KJ672454|RSVA|NA|F|2M|2013-03-08|USA|TN|	ON1	NA
KU950617|RSVA|NA|M|3W|2012-12-19|USA|USA|	ON1	NA
KJ672444|RSVA|NA|F|3M|2012-12-13|USA|TN|	ON1	NA
KU950654|RSVA|NA|F|9W|2012-09-11|USA|USA|	ON1	NA
KJ672467|RSVA|NA|F|3M|2013-01-31|USA|TN|	ON1	NA
KU950694|RSVA|NA|F|2W|2012-10-22|USA|USA|	ON1	NA
KU950475|RSVA|NA|M|8W|2012-09-21|USA|USA|	ON1	NA
KU950483|RSVA|NA|F|2W|2013-08-20|USA|USA|	ON1	NA
KU950521|RSVA|NA|M|8W|2012-08-15|USA|USA|	ON1	NA
KJ672432|RSVA|NA|F|3M|2013-03-11|USA|TN|	ON1	NA
KJ672448|RSVA|NA|F|1M|2013-03-08|USA|TN|	ON1	NA
JX627336|RSVA|NA|NA|NA|2011-12-27|KOR|KOR|	ON1	NA
KU950666|RSVA|NA|F|2W|2013-11-04|USA|USA|	ON1	NA
KJ672472|RSVA|NA|F|1M|2012-12-05|USA|TN|	ON1	NA
KU950651|RSVA|NA|F|2W|2012-11-12|USA|USA|	ON1	NA
KT285064|RSVA|ON1|NA|NA|2014-12-29|CHN|CHN|	ON1	NA
KJ672471|RSVA|NA|F|3M|2013-03-01|USA|TN|	ON1	NA
KU950610|RSVA|NA|F|17W|2013-01-07|USA|USA|	ON1	NA
KJ672428|RSVA|NA|M|2M|2013-01-24|USA|TN|	ON1	NA
KU950664|RSVA|NA|F|8W|2012-10-29|USA|USA|	ON1	NA
KJ672449|RSVA|NA|F|1M|2012-12-10|USA|TN|	ON1	NA
KU950557|RSVA|NA|F|7W|2012-08-08|USA|USA|	ON1	NA
KU950570|RSVA|NA|F|7W|2012-08-08|USA|USA|	ON1	NA
KU950550|RSVA|NA|F|10W|2012-10-02|USA|USA|	ON1	NA
61951|RSVA|NA|NA|NA|2013-02-17|JOR|Amman|	ON1	NA
KU950615|RSVA|NA|M|3W|2012-12-17|USA|USA|	ON1	NA
KU950631|RSVA|NA|M|7W|2012-09-21|USA|USA|	ON1	NA
61955|RSVA|NA|NA|NA|2012-10-23|JOR|Amman|	ON1	NA
61954|RSVA|NA|NA|NA|2013-01-16|JOR|Amman|	ON1	NA
KC731482|RSVA|NA|NA|NA|2011--|IND|IND|	ON1	NA

KU950594|RSVA|NA|M|8W|2013-12-20|USA|USA|	GA2	NA
KU950670|RSVA|NA|M|1W|2012-11-19|USA|USA|	GA2	NA
KM042391|RSVA|NA|F|7M|2013-03-15|USA|TN|	GA2	NA
KJ672437|RSVA|NA|F|1M|2013-01-11|USA|TN|	GA2	NA
KU950628|RSVA|NA|F|9W|2013-10-02|USA|USA|	GA2	NA
KU950636|RSVA|NA|F|8W|2012-11-01|USA|USA|	GA2	NA
KJ672457|RSVA|NA|M|5M|2013-01-07|USA|TN|	GA2	NA
KU950581|RSVA|NA|F|4W|2012-11-08|USA|USA|	GA2	NA
KU950583|RSVA|NA|F|2W|2013-07-12|USA|USA|	GA2	NA
KU950685|RSVA|NA|F|9W|2013-09-27|USA|USA|	GA2	NA
KU950459|RSVA|NA|M|9W|2013-12-19|USA|USA|	GA2	NA
KU950560|RSVA|NA|F|6W|2013-11-05|USA|USA|	GA2	NA
KU950540|RSVA|NA|M|22W|2013-11-26|USA|USA|	GA2	NA
KU950523|RSVA|NA|M|27W|2014-03-26|USA|USA|	GA2	NA
KU950486|RSVA|NA|M|18W|2012-11-20|USA|USA|	GA2	NA
KU950677|RSVA|NA|F|9W|2012-09-11|USA|USA|	GA2	NA
KU950537|RSVA|NA|F|6W|2014-01-08|USA|USA|	GA2	NA
KU950686|RSVA|NA|F|24W|2014-01-10|USA|USA|	GA2	NA
KU950506|RSVA|NA|M|8W|2013-10-10|USA|USA|	GA2	NA
KU950499|RSVA|NA|M|2W|2013-10-04|USA|USA|	GA2	NA
KU950520|RSVA|NA|M|2W|2013-06-20|USA|USA|	GA2	NA
KU950629|RSVA|NA|M|2W|2013-07-11|USA|USA|	GA2	NA
KU950643|RSVA|NA|F|6W|2012-10-02|USA|USA|	GA2	NA
KU950556|RSVA|NA|F|11W|2012-12-04|USA|USA|	GA2	NA
KU950655|RSVA|NA|M|17W|2012-12-27|USA|USA|	GA2	NA
KJ672458|RSVA|NA|F|2M|2013-02-18|USA|TN|	GA2	NA
KM042392|RSVA|NA|M|1M|2013-02-22|USA|TN|	GA2	NA
KJ672465|RSVA|NA|F|5M|2012-12-31|USA|TN|	GA2	NA
KU950531|RSVA|NA|F|2W|2013-08-19|USA|USA|	GA2	NA
KJ627264|RSVA|NA|M|12D|2012-05-16|PER|PER|	GA2	NA
KU950502|RSVA|NA|M|4W|2012-09-24|USA|USA|	GA2	NA
KU839626|RSVA|NA|NA|NA|2013-11-05|USA|TN|	GA2	NA
KU950544|RSVA|NA|F|7W|2013-12-17|USA|USA|	GA2	NA
KU950639|RSVA|NA|M|1W|2013-10-17|USA|USA|	GA2	NA
KU950491|RSVA|NA|M|6W|2013-09-04|USA|USA|	GA2	NA
KU839639|RSVA|NA|NA|NA|2013-12-17|USA|TN|	GA2	NA
KU950641|RSVA|NA|M|17W|2013-11-18|USA|USA|	GA2	NA
KU950650|RSVA|NA|F|14W|2013-09-23|USA|USA|	GA2	NA
KU839630|RSVA|NA|NA|NA|2014-01-07|USA|TN|	GA2	NA
KU950455|RSVA|NA|F|5W|2013-09-24|USA|USA|	GA2	NA

KU950505|RSVA|NA|M|4W|2013-11-07|USA|USA|	GA2	NA
KU950623|RSVA|NA|M|2W|2013-08-14|USA|USA|	GA2	NA
KU950566|RSVA|NA|M|18W|2013-11-26|USA|USA|	GA2	NA
KU950638|RSVA|NA|F|5W|2013-11-26|USA|USA|	GA2	NA
KU950644|RSVA|NA|M|2W|2013-11-25|USA|USA|	GA2	NA
KU839624|RSVA|NA|NA|NA|2013-11-11|USA|TN|	GA2	NA
KU950519|RSVA|NA|M|20W|2013-10-29|USA|USA|	GA2	NA
KU950549|RSVA|NA|M|10W|2013-11-05|USA|USA|	GA2	NA
KU950698|RSVA|NA|M|7W|2013-11-20|USA|USA|	GA2	NA
KU950518|RSVA|NA|M|17W|2013-10-09|USA|USA|	GA2	NA
KU839631|RSVA|NA|NA|NA|2013-11-26|USA|TN|	GA2	NA
KU950683|RSVA|NA|M|2W|2013-09-27|USA|USA|	GA2	NA
KU950527|RSVA|NA|F|9W|2013-08-26|USA|USA|	GA2	NA
KU950464|RSVA|NA|F|12W|2014-02-19|USA|USA|	GA2	NA
KU950456|RSVA|NA|M|2W|2013-08-07|USA|USA|	GA2	NA
KU950460|RSVA|NA|F|14W|2013-12-05|USA|USA|	GA2	NA
KU950590|RSVA|NA|M|1W|2013-07-24|USA|USA|	GA2	NA
KU950470|RSVA|NA|F|8W|2013-10-09|USA|USA|	GA2	NA
KU950608|RSVA|NA|M|7W|2013-10-15|USA|USA|	GA2	NA
KU950472|RSVA|NA|M|2W|2013-07-18|USA|USA|	GA2	NA
KU950598|RSVA|NA|M|2W|2013-10-07|USA|USA|	GA2	NA
KU950541|RSVA|NA|F|2W|2013-01-04|USA|USA|	GA2	NA
KU950595|RSVA|NA|M|1W|2012-12-21|USA|USA|	GA2	NA
KU950634|RSVA|NA|F|9W|2012-09-17|USA|USA|	GA2	NA
KJ672439|RSVA|NA|M|1M|2012-12-17|USA|TN|	GA2	NA
KU950658|RSVA|NA|M|3W|2012-09-12|USA|USA|	GA2	NA
KU950645|RSVA|NA|M|13W|2013-01-17|USA|USA|	GA2	NA
KU950579|RSVA|NA|F|9W|2012-10-09|USA|USA|	GA2	NA
KU950545|RSVA|NA|M|8W|2012-10-18|USA|USA|	GA2	NA
KJ672443|RSVA|NA|M|1M|2013-01-31|USA|TN|	GA2	NA
KJ672431|RSVA|NA|M|1M|2012-12-10|USA|TN|	GA2	NA
KJ672455|RSVA|NA|M|2M|2012-12-26|USA|TN|	GA2	NA
KU950538|RSVA|NA|M|18W|2012-10-25|USA|USA|	GA2	NA
KU950554|RSVA|NA|M|1W|2012-11-02|USA|USA|	GA2	NA
KU950522|RSVA|NA|F|9W|2012-11-13|USA|USA|	GA2	NA
KM042381|RSVA|NA|F|5M|2013-01-10|USA|TN|	GA2	NA
KJ672456|RSVA|NA|M|2M|2012-12-07|USA|TN|	GA2	NA
KJ672434|RSVA|NA|F|3M|2013-01-24|USA|TN|	GA2	NA
KJ672446|RSVA|NA|M|3M|2012-12-11|USA|TN|	GA2	NA
KU950528|RSVA|NA|M|26W|2013-01-14|USA|USA|	GA2	NA

KU950624|RSVA|NA|M|4W|2013-01-23|USA|USA|	GA2	NA
KJ672450|RSVA|NA|M|5M|2013-01-31|USA|TN|	GA2	NA
KU950546|RSVA|NA|M|9W|2012-09-14|USA|USA|	GA2	NA
KJ672442|RSVA|NA|M|1M|2013-02-11|USA|TN|	GA2	NA
KJ672435|RSVA|NA|M|2M|2012-12-28|USA|TN|	GA2	NA
KJ672426|RSVA|NA|M|3M|2013-01-08|USA|TN|	GA2	NA
KJ672478|RSVA|NA|F|4M|2013-01-10|USA|TN|	GA2	NA
KJ672477|RSVA|NA|M|2M|2012-12-20|USA|TN|	GA2	NA
KJ672427|RSVA|NA|F|6M|2012-11-09|USA|TN|	GA2	NA
KJ672484|RSVA|NA|M|4M|2012-12-26|USA|TN|	GA2	NA
KJ672482|RSVA|NA|M|2M|2012-12-28|USA|TN|	GA2	NA
KU950498|RSVA|NA|M|7W|2012-10-04|USA|USA|	GA2	NA
KM042385|RSVA|NA|F|1M|2013-01-07|USA|TN|	GA2	NA
KU950469|RSVA|NA|M|3W|2012-09-11|USA|USA|	GA2	NA
KU950632|RSVA|NA|M|7W|2012-10-19|USA|USA|	GA2	NA
KU950620|RSVA|NA|M|17W|2012-10-04|USA|USA|	GA2	NA
KU950563|RSVA|NA|M|17W|2012-10-29|USA|USA|	GA2	NA
KU950529|RSVA|NA|M|17W|2012-09-28|USA|USA|	GA2	NA
KJ672460|RSVA|NA|M|1M|2013-01-04|USA|TN|	GA2	NA
KJ672463|RSVA|NA|F|1M|2013-01-08|USA|TN|	GA2	NA
KM042384|RSVA|NA|M|3M|2013-02-13|USA|TN|	GA2	NA
KU950646|RSVA|NA|M|1W|2012-11-21|USA|USA|	GA2	NA
KJ672453|RSVA|NA|M|5M|2013-01-10|USA|TN|	GA2	NA
KU950507|RSVA|NA|M|3W|2012-08-27|USA|USA|	GA2	NA
KU950536|RSVA|NA|F|10W|2012-08-16|USA|USA|	GA2	NA
KU950649|RSVA|NA|F|10W|2012-08-16|USA|USA|	GA2	NA
KU950667|RSVA|NA|F|16W|2012-10-05|USA|USA|	GA2	NA
KJ672459|RSVA|NA|M|4M|2013-01-23|USA|TN|	GA2	NA
KU950661|RSVA|NA|M|21W|2012-12-26|USA|USA|	GA2	NA
KJ672436|RSVA|NA|F|6M|2012-12-06|USA|TN|	GA2	NA
61936|RSVA|NA|NA|NA|2011-03-01|JOR|Amman|	GA2	NA
61975|RSVA|NA|NA|NA|2012-01-08|JOR|Amman|	GA2	NA
KJ627374|RSVA|NA|F|1Y|2011-04-19|PER|PER|	GA2	NA
KU950652|RSVA|NA|M|9W|2012-09-10|USA|USA|	GA2	NA
JF920053|RSVA|GA2|NA|NA|2010-02-16|USA|WI|	GA2	NA
KF826849|RSVA|NA|F|2Y|2010-01-05|USA|WI|	GA2	NA
KF530269|RSVA|NA|M|2Y|2009-12-10|USA|WI|	GA2	NA
JF920052|RSVA|GA2|NA|NA|2010-01-13|USA|WI|	GA2	NA
61995|RSVA|NA|NA|NA|2012-03-19|JOR|Amman|	GA2	12
61999|RSVA|NA|NA|NA|2012-03-06|JOR|Amman|	GA2	12

61962|RSVA|NA|NA|NA|2011-02-08|JOR|Amman|	GA2	5
KC731483|RSVA|NA|NA|NA|2011--|IND|IND|	GA2	NA
KP663728|RSVA|NA|NA|NA|2012-07-16|KOR|KOR|	GA2	NA
KC978856|RSVA|NA|NA|NA|2011-12-11|CHN|CHN|	GA2	NA
61944|RSVA|NA|NA|NA|2012-12-31|JOR|Amman|	GA2	14
KJ627292|RSVA|NA|F|9M|2010-04-19|PER|PER|	GA2	NA
KJ627371|RSVA|NA|M|14Y|2010-05-01|PER|PER|	GA2	NA
KJ627358|RSVA|NA|M|10M|2010-03-05|PER|PER|	GA2	NA
KJ627263|RSVA|NA|M|10M|2010-08-01|PER|PER|	GA2	NA
KJ627334|RSVA|NA|F|6Y|2010-08-06|PER|PER|	GA2	NA
KJ627296|RSVA|NA|F|10Y|2010-04-24|PER|PER|	GA2	NA
KJ627345|RSVA|NA|M|1M|2010-03-19|PER|PER|	GA2	NA
KJ627357|RSVA|NA|M|2Y|2010-04-30|PER|PER|	GA2	NA
KJ627307|RSVA|NA|M|1Y|2010-05-25|PER|PER|	GA2	NA
KJ627273|RSVA|NA|F|6M|2010-03-16|PER|PER|	GA2	NA
KJ627269|RSVA|NA|M|1M|2010-01-30|PER|PER|	GA2	NA
KJ627270|RSVA|NA|M|4M|2010-03-28|PER|PER|	GA2	NA
KJ627329|RSVA|NA|M|7Y|2010-04-28|PER|PER|	GA2	NA
KJ627313|RSVA|NA|F|21D|2010-04-22|PER|PER|	GA2	NA
KJ627298|RSVA|NA|M|2Y|2010-04-14|PER|PER|	GA2	NA
KJ627325|RSVA|NA|F|1Y|2010-03-30|PER|PER|	GA2	NA
KJ627258|RSVA|NA|M|4Y|2010-04-24|PER|PER|	GA2	NA
KJ627333|RSVA|NA|F|2M|2010-03-11|PER|PER|	GA2	NA
KF826833|RSVA|NA|M|1Y|2009-03-19|ITA|ITA|	GA2	
61932|RSVA|NA|NA|NA|2010-12-26|JOR|Amman|	GA2	3
61927|RSVA|NA|NA|NA|2011-04-18|JOR|Amman|	GA2	3
61961|RSVA|NA|NA|NA|2011-02-06|JOR|Amman|	GA2	3
61928|RSVA|NA|NA|NA|2011-03-20|JOR|Amman|	GA2	3
61963|RSVA|NA|NA|NA|2011-02-09|JOR|Amman|	GA2	3
61947|RSVA|NA|NA|NA|2011-02-27|JOR|Amman|	GA2	3
61935|RSVA|NA|NA|NA|2011-03-02|JOR|Amman|	GA2	3
62014|RSVA|NA|NA|NA|2011-02-21|JOR|Amman|	GA2	3
61929|RSVA|NA|NA|NA|2011-01-24|JOR|Amman|	GA2	3
61934|RSVA|NA|NA|NA|2011-01-24|JOR|Amman|	GA2	3
61990|RSVA|NA|NA|NA|2012-02-05|JOR|Amman|	GA2	3
61978|RSVA|NA|NA|NA|2012-02-12|JOR|Amman|	GA2	11
61968|RSVA|NA|NA|NA|2012-02-16|JOR|Amman|	GA2	4
61976|RSVA|NA|NA|NA|2012-02-05|JOR|Amman|	GA2	4
61997|RSVA|NA|NA|NA|2012-02-21|JOR|Amman|	GA2	4
61998|RSVA|NA|NA|NA|2012-02-20|JOR|Amman|	GA2	4

61988|RSVA|NA|NA|NA|2012-02-01|JOR|Amman|	GA2	4
61974|RSVA|NA|NA|NA|2012-01-22|JOR|Amman|	GA2	4
62002|RSVA|NA|NA|NA|2012-02-28|JOR|Amman|	GA2	4
61984|RSVA|NA|NA|NA|2012-01-26|JOR|Amman|	GA2	4
62016|RSVA|NA|NA|NA|2012-01-26|JOR|Amman|	GA2	4
61960|RSVA|NA|NA|NA|2011-02-02|JOR|Amman|	GA2	4
61930|RSVA|NA|NA|NA|2011-01-26|JOR|Amman|	GA2	4
KF826855|RSVA|NA|F|1M|2009-03-26|ITA|ITA|	GA2	NA
61965|RSVA|NA|NA|NA|2010-03-22|JOR|Amman|	GA2	1
61979|RSVA|NA|NA|NA|2010-05-05|JOR|Amman|	GA2	1
61977|RSVA|NA|NA|NA|2010-06-15|JOR|Amman|	GA2	1
61926|RSVA|NA|NA|NA|2011-03-14|JOR|Amman|	GA2	6
KJ627316|RSVA|NA|F|4M|2010-04-21|PER|PER|	GA2	NA
KM517572|RSVA|NA|NA|NA|2011-02-24|CHN|CHN|	GA2	NA
JX015496|RSVA|GA2|NA|NA|2008-11-26|NLD|NLD|	GA2	NA
62015|RSVA|NA|NA|NA|2011-03-20|JOR|Amman|	GA2	7
KJ627351|RSVA|NA|M|2Y|2011-04-13|PER|PER|	GA2	NA
KJ627370|RSVA|NA|M|1Y|2011-05-16|PER|PER|	GA2	NA
KJ627320|RSVA|NA|F|4Y|2010-04-24|PER|PER|	GA2	NA
KJ627250|RSVA|NA|M|2Y|2010-06-08|PER|PER|	GA2	NA
KJ627252|RSVA|NA|F|3Y|2010-01-19|PER|PER|	GA2	NA
KJ627297|RSVA|NA|F|2Y|2010-05-07|PER|PER|	GA2	NA
KJ627312|RSVA|NA|F|6Y|2010-05-20|PER|PER|	GA2	NA
KJ627283|RSVA|NA|F|4Y|2010-05-15|PER|PER|	GA2	NA
KJ627355|RSVA|NA|F|1Y|2010-02-17|PER|PER|	GA2	NA
KJ627295|RSVA|NA|F|2Y|2010-02-24|PER|PER|	GA2	NA
KJ627346|RSVA|NA|F|1Y|2010-02-28|PER|PER|	GA2	NA
KJ627326|RSVA|NA|M|3Y|2010-02-23|PER|PER|	GA2	NA
KJ627287|RSVA|NA|M|7M|2010-04-08|PER|PER|	GA2	NA
KJ627268|RSVA|NA|F|4Y|2010-04-22|PER|PER|	GA2	NA
KJ627261|RSVA|NA|M|3Y|2010-04-21|PER|PER|	GA2	NA
KJ627301|RSVA|NA|F|8M|2010-04-14|PER|PER|	GA2	NA
KJ627245|RSVA|NA|M|1M|2010-04-29|PER|PER|	GA2	NA
KJ627309|RSVA|NA|F|10Y|2010-04-15|PER|PER|	GA2	NA
KJ627279|RSVA|NA|F|6Y|2010-05-18|PER|PER|	GA2	NA
KJ627367|RSVA|NA|M|5Y|2010-05-06|PER|PER|	GA2	NA
KJ627253|RSVA|NA|M|2M|2009-09-25|PER|PER|	GA2	NA
KJ627372|RSVA|NA|F|7M|2010-02-27|PER|PER|	GA2	NA
KJ627256|RSVA|NA|M|10M|2009-08-16|PER|PER|	GA2	NA
KJ627365|RSVA|NA|M|1Y|2010-03-23|PER|PER|	GA2	NA

KJ627306|RSVA|NA|F|4M|2009-05-11|PER|PER|	GA2	NA
KJ627360|RSVA|NA|M|2Y|2009-05-14|PER|PER|	GA2	NA
KJ627318|RSVA|NA|F|1Y|2009-04-17|PER|PER|	GA2	NA
KJ627291|RSVA|NA|M|4Y|2009-05-02|PER|PER|	GA2	NA
KJ627373|RSVA|NA|M|4Y|2009-05-13|PER|PER|	GA2	NA
KJ627315|RSVA|NA|M|11M|2009-05-22|PER|PER|	GA2	NA
KJ627272|RSVA|NA|F|2M|2009-04-27|PER|PER|	GA2	NA
KJ627319|RSVA|NA|F|1Y|2009-04-21|PER|PER|	GA2	NA
KJ627344|RSVA|NA|F|4Y|2009-04-23|PER|PER|	GA2	NA
KJ627286|RSVA|NA|M|8M|2009-04-18|PER|PER|	GA2	NA
KJ627350|RSVA|NA|M|2Y|2009-04-05|PER|PER|	GA2	NA
KJ627294|RSVA|NA|F|45Y|2010-05-20|PER|PER|	GA2	NA
61971|RSVA|NA|NA|NA|2012-01-30|JOR|Amman|	GA2	9
61993|RSVA|NA|NA|NA|2012-02-21|JOR|Amman|	GA2	9
KJ939944|RSVA|NA|NA|NA|2010-06-22|VNM|VNM|	GA2	NA
KJ939963|RSVA|NA|NA|NA|2010-08-09|VNM|VNM|	GA2	NA
KJ939960|RSVA|NA|NA|NA|2010-07-09|VNM|VNM|	GA2	NA
KJ939950|RSVA|NA|NA|NA|2010-07-02|VNM|VNM|	GA2	NA
KJ939956|RSVA|NA|NA|NA|2010-05-05|VNM|VNM|	GA2	NA
KJ939949|RSVA|NA|NA|NA|2010-03-26|VNM|VNM|	GA2	NA
KJ939957|RSVA|NA|NA|NA|2010-05-17|VNM|VNM|	GA2	NA
KJ939952|RSVA|NA|NA|NA|2009-11-11|VNM|VNM|	GA2	NA
KJ939962|RSVA|NA|NA|NA|2010-07-13|VNM|VNM|	GA2	NA
KJ939964|RSVA|NA|NA|NA|2010-08-12|VNM|VNM|	GA2	NA
KJ939959|RSVA|NA|NA|NA|2010-06-30|VNM|VNM|	GA2	NA
KJ939969|RSVA|NA|NA|NA|2010-09-23|VNM|VNM|	GA2	NA
KJ939945|RSVA|NA|NA|NA|2010-06-23|VNM|VNM|	GA2	NA
KJ939958|RSVA|NA|NA|NA|2010-06-15|VNM|VNM|	GA2	NA
KJ939941|RSVA|NA|NA|NA|2010-04-21|VNM|VNM|	GA2	NA
KJ939951|RSVA|NA|NA|NA|2009-10-28|VNM|VNM|	GA2	NA
KJ939968|RSVA|NA|NA|NA|2010-09-15|VNM|VNM|	GA2	NA
KJ939967|RSVA|NA|NA|NA|2010-09-15|VNM|VNM|	GA2	NA
KJ939946|RSVA|NA|NA|NA|2010-07-05|VNM|VNM|	GA2	NA
KJ939953|RSVA|NA|NA|NA|2010-03-18|VNM|VNM|	GA2	NA
KJ939942|RSVA|NA|NA|NA|2010-06-04|VNM|VNM|	GA2	NA
JX015479|RSVA|GA2|NA|NA|2011-01-03|NLD|NLD|	GA2	NA
KJ627303|RSVA|NA|F|2M|2010-04-20|PER|PER|	GA2	NA
KJ627308|RSVA|NA|F|2Y|2010-04-13|PER|PER|	GA2	NA
KJ627343|RSVA|NA|M|4Y|2010-03-30|PER|PER|	GA2	NA
KJ627356|RSVA|NA|M|1Y|2010-04-22|PER|PER|	GA2	NA

KM578843|RSVA|NA|NA|NA|2012-01-10|CHN|CHN|	GA2	NA
JX015483|RSVA|GA2|NA|NA|2008-12-29|NLD|NLD|	GA2	NA
JF920054|RSVA|GA2|NA|NA|2010-02-16|USA|WI|	GA2	NA
KJ627349|RSVA|NA|M|1M|2009-09-17|PER|PER|	GA2	NA
KJ627246|RSVA|NA|M|3M|2009-04-23|PER|PER|	GA2	NA
KJ627304|RSVA|NA|M|2Y|2009-04-15|PER|PER|	GA2	NA
KJ627322|RSVA|NA|M|9M|2009-04-03|PER|PER|	GA2	NA
KJ627300|RSVA|NA|M|1Y|2009-05-13|PER|PER|	GA2	NA
KJ939955|RSVA|NA|NA|NA|2010-04-29|VNM|VNM|	GA2	NA
KJ939970|RSVA|NA|NA|NA|2010-11-29|VNM|VNM|	GA2	NA
61986|RSVA|NA|NA|NA|2012-01-31|JOR|Amman|	GA2	10
61994|RSVA|NA|NA|NA|2012-03-29|JOR|Amman|	GA2	13
KJ627259|RSVA|NA|M|7Y|2011-04-08|PER|PER|	GA2	NA
KF826856|RSVA|NA|M|9Y|2009-04-23|ITA|ITA|	GA2	NA
61972|RSVA|NA|NA|NA|2012-01-09|JOR|Amman|	GA2	2
61980|RSVA|NA|NA|NA|2012-01-23|JOR|Amman|	GA2	2
62004|RSVA|NA|NA|NA|2012-04-12|JOR|Amman|	GA2	2
61969|RSVA|NA|NA|NA|2012-01-31|JOR|Amman|	GA2	2
62007|RSVA|NA|NA|NA|2013-03-26|JOR|Amman|	GA2	2
61937|RSVA|NA|NA|NA|2011-03-08|JOR|Amman|	GA2	2
61933|RSVA|NA|NA|NA|2011-01-19|JOR|Amman|	GA2	2
62013|RSVA|NA|NA|NA|2011-02-20|JOR|Amman|	GA2	2
61991|RSVA|NA|NA|NA|2012-03-13|JOR|Amman|	GA2	2
61996|RSVA|NA|NA|NA|2012-04-04|JOR|Amman|	GA2	2
61970|RSVA|NA|NA|NA|2012-02-12|JOR|Amman|	GA2	2
61985|RSVA|NA|NA|NA|2012-02-09|JOR|Amman|	GA2	2
62000|RSVA|NA|NA|NA|2012-02-28|JOR|Amman|	GA2	2
KJ939937|RSVA|NA|NA|NA|2009-08-12|VNM|VNM|	GA2	NA
KJ939938|RSVA|NA|NA|NA|2009-09-11|VNM|VNM|	GA2	NA
KJ939940|RSVA|NA|NA|NA|2009-12-01|VNM|VNM|	GA2	NA
KJ939935|RSVA|NA|NA|NA|2009-07-21|VNM|VNM|	GA2	NA
KJ939936|RSVA|NA|NA|NA|2009-07-22|VNM|VNM|	GA2	NA
KJ939939|RSVA|NA|NA|NA|2009-10-15|VNM|VNM|	GA2	NA
KJ939965|RSVA|NA|NA|NA|2010-08-24|VNM|VNM|	GA2	NA
KJ627267|RSVA|NA|M|6M|2010-02-08|PER|PER|	GA2	NA
KJ627275|RSVA|NA|F|5Y|2010-05-24|PER|PER|	GA2	NA
JX015494|RSVA|GA2|NA|NA|2008-01-14|NLD|NLD|	GA2	NA
KF530261|RSVA|NA|NA|NA|2008-05-01|DEU|DEU|	GA2	NA
KP317953|RSVA|NA|NA|NA|2012-03-23|KEN|KEN|	GA2	NA
61987|RSVA|NA|NA|NA|2012-01-17|JOR|Amman|	GA2	8

61989|RSVA|NA|NA|NA|2012-01-30|JOR|Amman|	GA2	8
62001|RSVA|NA|NA|NA|2012-02-27|JOR|Amman|	GA2	8
61983|RSVA|NA|NA|NA|2011-12-19|JOR|Amman|	GA2	8
KJ627271|RSVA|NA|F|1Y|2009-05-29|PER|PER|	GA2	NA
KJ939961|RSVA|NA|NA|NA|2010-07-09|VNM|VNM|	GA2	NA
KJ939971|RSVA|NA|NA|NA|2010-12-06|VNM|VNM|	GA2	NA
JX015484|RSVA|GA2|NA|NA|2007-11-06|NLD|NLD|	GA2	NA
JX015492|RSVA|GA2|NA|NA|2007-12-24|NLD|NLD|	GA2	NA
JX015489|RSVA|GA2|NA|NA|2007-12-03|NLD|NLD|	GA2	NA
JX015491|RSVA|GA2|NA|NA|2007-11-23|NLD|NLD|	GA2	NA
JX015490|RSVA|GA2|NA|NA|2007-11-19|NLD|NLD|	GA2	NA
JX015493|RSVA|GA2|NA|NA|2008-01-07|NLD|NLD|	GA2	NA
JX015482|RSVA|GA2|NA|NA|2006-11-22|BEL|BEL|	GA2	NA
KF826831|RSVA|NA|NA|NA|2009-12-01|DEU|DEU|	GA2	NA
KJ939947|RSVA|NA|NA|NA|2010-07-22|VNM|VNM|	GA2	NA
KJ939966|RSVA|NA|NA|NA|2010-09-03|VNM|VNM|	GA2	NA
JX015498|RSVA|GA2|NA|NA|2008-12-27|NLD|NLD|	GA2	NA
KF826830|RSVA|NA|NA|NA|2009-03-01|DEU|DEU|	GA2	NA
KJ627328|RSVA|NA|M|2Y|2010-05-20|PER|PER|	GA2	NA
KJ627369|RSVA|NA|M|2Y|2009-03-18|PER|PER|	GA2	NA
KJ627281|RSVA|NA|F|11M|2009-04-14|PER|PER|	GA2	NA
KJ627354|RSVA|NA|F|4Y|2009-03-25|PER|PER|	GA2	NA
KJ627338|RSVA|NA|F|4M|2009-05-08|PER|PER|	GA2	NA
KJ627293|RSVA|NA|F|5M|2009-05-25|PER|PER|	GA2	NA
JF920046|RSVA|GA2|NA|NA|2007--|USA|WI|	GA2	NA
JF920047|RSVA|GA2|NA|NA|2008-01-17|USA|WI|	GA2	NA
JF920048|RSVA|GA2|NA|NA|2007--|USA|WI|	GA2	NA
KF826821|RSVA|NA|NA|NA|2007--|USA|WI|	GA2	NA
KJ627266|RSVA|NA|M|2Y|2009-04-13|PER|PER|	GA2	NA
KJ627288|RSVA|NA|F|1Y|2009-05-28|PER|PER|	GA2	NA
KJ627274|RSVA|NA|M|5M|2009-04-02|PER|PER|	GA2	NA
KJ627362|RSVA|NA|M|1Y|2009-05-19|PER|PER|	GA2	NA
KJ627282|RSVA|NA|M|6M|2009-04-08|PER|PER|	GA2	NA
KF826840|RSVA|NA|F|2M|2007-02-16|MEX|MEX|	GA2	NA
JF920049|RSVA|GA2|NA|NA|2007-12-28|USA|WI|	GA2	NA
JF920051|RSVA|GA2|NA|NA|2007-12-22|USA|WI|	GA2	NA
JX015499|RSVA|GA2|NA|NA|2008-10-22|BEL|BEL|	GA2	NA
KJ627337|RSVA|NA|F|1Y|2009-05-27|PER|PER|	GA2	NA
KJ627335|RSVA|NA|F|4M|2009-05-16|PER|PER|	GA2	NA
KJ627290|RSVA|NA|M|1Y|2009-03-23|PER|PER|	GA2	NA

KJ627257|RSVA|NA|M|NA|2009-02-09|PER|PER|	GA2	NA
KF826838|RSVA|NA|NA|NA|2006-06-06|ARG|ARG|	GA2	NA
JX015495|RSVA|GA2|NA|NA|2008-11-21|NLD|NLD|	GA2	NA
JX015497|RSVA|GA2|NA|NA|2008-12-06|NLD|NLD|	GA2	NA
JQ901447|RSVA|GA2|NA|NA|2001-01-03|NLD|NLD|	GA2	NA
KJ627324|RSVA|NA|F|11Y|2009-04-20|PER|PER|	GA2	NA
KJ627321|RSVA|NA|M|4Y|2009-04-17|PER|PER|	GA2	NA
KJ627366|RSVA|NA|F|10M|2009-04-20|PER|PER|	GA2	NA
KJ627339|RSVA|NA|M|2Y|2009-03-11|PER|PER|	GA2	NA
KJ627314|RSVA|NA|F|9M|2009-05-18|PER|PER|	GA2	NA
KJ627284|RSVA|NA|M|3Y|2009-05-29|PER|PER|	GA2	NA
KJ627289|RSVA|NA|M|2Y|2009-05-11|PER|PER|	GA2	NA
KJ627248|RSVA|NA|F|6Y|2009-04-30|PER|PER|	GA2	NA
KJ627276|RSVA|NA|F|3Y|2009-04-29|PER|PER|	GA2	NA
KJ627327|RSVA|NA|F|1M|2007-07-25|PER|PER|	GA2	NA
KJ627260|RSVA|NA|M|4Y|2008-03-17|PER|PER|	GA2	NA
KJ627336|RSVA|NA|F|3M|2008-02-29|PER|PER|	GA2	NA
KJ627255|RSVA|NA|F|1M|2009-04-01|PER|PER|	GA2	NA
KJ627311|RSVA|NA|F|4M|2008-06-06|PER|PER|	GA2	NA
KJ627353|RSVA|NA|F|1M|2008-03-18|PER|PER|	GA2	NA
JX069799|RSVA|GA2|F|NA|2001-03-13|USA|TN|	GA2	NA
JQ901457|RSVA|GA2|NA|NA|2003-12-22|NLD|NLD|	GA2	NA
JX015486|RSVA|GA2|NA|NA|2005-01-05|NLD|NLD|	GA2	NA
JQ901458|RSVA|GA2|NA|NA|2003-12-23|NLD|NLD|	GA2	NA
JF920057|RSVA|GA2|NA|NA|1998-03-21|USA|WI|	GA2	NA
KP856968|RSVA|NA|M|26M|1998--|USA|NY|	GA2	NA
KU316141|RSVA|NA|M|7M|1998--|USA|NY|	GA2	NA
KU316091|RSVA|NA|F|3M|1998--|USA|NY|	GA2	NA
KF826848|RSVA|NA|NA|NA|2007--|AUS|AUS|	GA2	NA
JX015480|RSVA|GA2|NA|NA|2007-12-10|NLD|NLD|	GA2	NA
JQ901452|RSVA|GA2|NA|NA|2001-12-22|NLD|NLD|	GA2	NA
JQ901453|RSVA|GA2|NA|NA|2002-01-03|NLD|NLD|	GA2	NA
KJ627352|RSVA|NA|M|3Y|2007-03-17|PER|PER|	GA2	NA
KJ627361|RSVA|NA|F|11M|2007-01-26|PER|PER|	GA2	NA
KJ627305|RSVA|NA|F|1Y|2007-03-17|PER|PER|	GA2	NA
KP119748|RSVA|NA|NA|NA|2012-09-20|HKG|HKG|	GA2	NA
KP119746|RSVA|NA|NA|NA|2012-09-20|HKG|HKG|	GA2	NA
KP119745|RSVA|NA|NA|NA|2012-09-20|HKG|HKG|	GA2	NA
KP119747|RSVA|NA|NA|NA|2012-09-20|HKG|HKG|	GA2	NA
KP218910|RSVA|NA|NA|NA|2008-11-30|CHN|CHN|	GA2	NA

KU950573|RSVA|NA|F|38W|2006-01-07|USA|USA|	GA2	NA
KJ627647|RSVA|NA|M|2M|2001--|USA|TN|	GA2	NA
KJ627648|RSVA|NA|M|2M|2001--|USA|TN|	GA2	NA
JX069798|RSVA|GA2|M|NA|2001-02-20|USA|TN|	GA2	NA
KP258697|RSVA|NA|F|1.2M|1994--|USA|NY|	GA2	NA
KU316139|RSVA|NA|F|12M|1994--|USA|NY|	GA2	NA
KU316131|RSVA|NA|F|2M|1994--|USA|NY|	GA2	NA
KU316118|RSVA|NA|M|4M|1996--|USA|NY|	GA2	NA
KU316126|RSVA|NA|F|20M|1984--|USA|NY|	GA2	NA
KU316142|RSVA|NA|F|11M|1982--|USA|NY|	GA2	NA
KU316148|RSVA|NA|F|3W|1984--|USA|NY|	GA2	NA
KU316166|RSVA|NA|F|1Y|1977--|USA|NY|	GA2	NA
JF920065|RSVA|GA7|NA|NA|1998-01-09|USA|WI|	GA7	NA
JX069801|RSVA|NA|M|NA|1998-03-02|USA|TN|	GA7	NA
JX069800|RSVA|NA|F|NA|1997-12-22|USA|TN|	GA7	NA
JF920062|RSVA|GA7|NA|NA|1998-01-06|USA|WI|	GA7	NA
KP258743|RSVA|NA|M|7M|1998--|USA|NY|	GA7	NA
KU316092|RSVA|NA|M|2Y|1991--|USA|NY|	GA7	NA
KP258737|RSVA|NA|M|1Y|1984--|USA|NY|	GA7	NA
KP258700|RSVA|NA|F|3Y|1985--|USA|NY|	GA7	NA
KU316167|RSVA|NA|F|5W|1985--|USA|NY|	GA7	NA
KU316110|RSVA|NA|M|6W|1984--|USA|NY|	GA7	NA
KJ723464|RSVA|NA|M|52W|1989--|USA|NY|	GA7	NA
KP258734|RSVA|NA|F|9M|1989--|USA|NY|	GA7	NA
KJ723492|RSVA|NA|M|3W|1990--|USA|NY|	GA7	NA
KJ723483|RSVA|NA|M|10W|1984--|USA|NY|	Undetermined genotype	NA
KU316098|RSVA|NA|F|2Y|1984--|USA|NY|	Undetermined genotype	NA
KU316125|RSVA|NA|M|5W|1984--|USA|NY|	Undetermined genotype	NA
KP258733|RSVA|NA|M|4Y|1984--|USA|NY|	Undetermined genotype	NA
KU316138|RSVA|NA|M|9M|1987--|USA|NY|	Undetermined genotype	NA
KP258723|RSVA|NA|F|3Y|1986--|USA|NY|	Undetermined genotype	NA
KJ672479|RSVA|NA|M|2M|2013-01-28|USA|TN|	GA5	NA
KJ672447|RSVA|NA|M|3M|2013-02-11|USA|TN|	GA5	NA
KJ672474|RSVA|NA|M|3M|2012-12-31|USA|TN|	GA5	NA
KU950564|RSVA|NA|F|9W|2012-09-12|USA|USA|	GA5	NA
KU950501|RSVA|NA|F|2W|2013-01-10|USA|USA|	GA5	NA
KU950473|RSVA|NA|F|17W|2012-12-04|USA|USA|	GA5	NA
KU950479|RSVA|NA|M|20W|2012-11-19|USA|USA|	GA5	NA
KU950487|RSVA|NA|F|10W|2012-11-07|USA|USA|	GA5	NA
KU950616|RSVA|NA|M|9W|2012-09-24|USA|USA|	GA5	NA

KU950609|RSVA|NA|M|18W|2012-10-22|USA|USA|	GA5	NA
KJ672462|RSVA|NA|M|2M|2013-03-04|USA|TN|	GA5	NA
KJ672483|RSVA|NA|M|1M|2013-01-30|USA|TN|	GA5	NA
KF826826|RSVA|NA|M|16M|2004-12-24|MEX|MEX|	GA5	NA
JX015487|RSVA|GA5|NA|NA|2006-01-02|NLD|NLD|	GA5	NA
KF826841|RSVA|NA|NA|NA|2007-05-02|ARG|ARG|	GA5	NA
KF826850|RSVA|NA|NA|NA|2008--|USA|WI|	GA5	NA
KF530268|RSVA|NA|M|25M|2007-01-27|MEX|MEX|	GA5	NA
KF826852|RSVA|NA|NA|NA|2007--|USA|WI|	GA5	NA
KF826836|RSVA|NA|M|1M|2006-01-04|MEX|MEX|	GA5	NA
KF826837|RSVA|NA|F|2M|2006-01-20|MEX|MEX|	GA5	NA
KF826846|RSVA|NA|NA|NA|2008-05-15|ARG|ARG|	GA5	NA
KF826828|RSVA|NA|NA|NA|2004-06-04|ARG|ARG|	GA5	NA
KJ627722|RSVA|NA|F|3M|2004--|USA|TN|	GA5	NA
KJ627721|RSVA|NA|F|3M|2004--|USA|TN|	GA5	NA
KJ627720|RSVA|NA|F|3M|2004--|USA|TN|	GA5	NA
KJ627719|RSVA|NA|M|1M|2004--|USA|TN|	GA5	NA
KJ627662|RSVA|NA|M|1M|2003--|USA|TN|	GA5	NA
KJ627714|RSVA|NA|M|7M|2003--|USA|TN|	GA5	NA
KJ627715|RSVA|NA|M|7M|2003--|USA|TN|	GA5	NA
KJ627668|RSVA|NA|M|0.6M|2003--|USA|TN|	GA5	NA
KJ627667|RSVA|NA|M|0.6M|2003--|USA|TN|	GA5	NA
KJ627666|RSVA|NA|M|0.6M|2003--|USA|TN|	GA5	NA
KJ627716|RSVA|NA|F|3M|2003--|USA|TN|	GA5	NA
KJ627718|RSVA|NA|F|3M|2003--|USA|TN|	GA5	NA
KJ627717|RSVA|NA|F|3M|2001--|USA|TN|	GA5	NA
KF826827|RSVA|NA|NA|NA|2004-05-26|ARG|ARG|	GA5	NA
KJ627729|RSVA|NA|F|0.5M|2003--|USA|TN|	GA5	NA
KF826832|RSVA|NA|M|3M|2009-03-19|ITA|ITA|	GA5	NA
JQ901449|RSVA|GA5|NA|NA|2001-01-10|NLD|NLD|	GA5	NA
JQ901448|RSVA|GA5|NA|NA|2001-01-07|NLD|NLD|	GA5	NA
JQ901456|RSVA|GA5|NA|NA|2003-11-25|NLD|NLD|	GA5	NA
KF973328|RSVA|NA|M|4M|2002--|USA|TN|	GA5	NA
KF973321|RSVA|NA|M|4M|2002--|USA|TN|	GA5	NA
KF973329|RSVA|NA|M|4M|2002--|USA|TN|	GA5	NA
KF973323|RSVA|NA|M|4M|2002--|USA|TN|	GA5	NA
KF973320|RSVA|NA|M|4M|2002--|USA|TN|	GA5	NA
KF973333|RSVA|NA|M|4M|2002--|USA|TN|	GA5	NA
KF973324|RSVA|NA|M|4M|2002--|USA|TN|	GA5	NA
KF973319|RSVA|NA|M|4M|2002--|USA|TN|	GA5	NA

KF973336|RSVA|NA|M|4M|2002--|USA|TN|	GA5	NA
KF973327|RSVA|NA|M|4M|2002--|USA|TN|	GA5	NA
KF973332|RSVA|NA|M|4M|2002--|USA|TN|	GA5	NA
KF973330|RSVA|NA|M|4M|2002--|USA|TN|	GA5	NA
KF973325|RSVA|NA|M|4M|2002--|USA|TN|	GA5	NA
KF973326|RSVA|NA|M|4M|2002--|USA|TN|	GA5	NA
KF973322|RSVA|NA|M|4M|2002--|USA|TN|	GA5	NA
KF973339|RSVA|NA|M|4M|2002--|USA|TN|	GA5	NA
KF973338|RSVA|NA|M|4M|2002--|USA|TN|	GA5	NA
KF973335|RSVA|NA|M|4M|2002--|USA|TN|	GA5	NA
KF973331|RSVA|NA|M|4M|2002--|USA|TN|	GA5	NA
KF973334|RSVA|NA|M|4M|2002--|USA|TN|	GA5	NA
KF973340|RSVA|NA|M|4M|2002--|USA|TN|	GA5	NA
KJ627649|RSVA|NA|F|3M|2001--|USA|TN|	GA5	NA
JQ901450|RSVA|GA5|NA|NA|2001-01-24|NLD|NLD|	GA5	NA
JQ901451|RSVA|GA5|NA|NA|2001-01-24|NLD|NLD|	GA5	NA
KJ627708|RSVA|NA|M|0.5M|2001--|USA|TN|	GA5	NA
KJ627709|RSVA|NA|M|0.5M|2001--|USA|TN|	GA5	NA
KJ627676|RSVA|NA|M|1M|2001--|USA|TN|	GA5	NA
KJ627663|RSVA|NA|F|1M|2003--|USA|TN|	GA5	NA
KJ627664|RSVA|NA|F|1M|2003--|USA|TN|	GA5	NA
KJ627670|RSVA|NA|M|0.4M|2003--|USA|TN|	GA5	NA
KJ627671|RSVA|NA|M|2M|2003--|USA|TN|	GA5	NA
KJ627657|RSVA|NA|M|4M|2003--|USA|TN|	GA5	NA
KJ627659|RSVA|NA|M|4M|2003--|USA|TN|	GA5	NA
KJ627724|RSVA|NA|M|0.3M|2004--|USA|TN|	GA5	NA
KJ627723|RSVA|NA|M|0.3M|2004--|USA|TN|	GA5	NA
KJ627725|RSVA|NA|M|0.3M|2004--|USA|TN|	GA5	NA
KJ627727|RSVA|NA|F|3M|2003--|USA|TN|	GA5	NA
KJ627728|RSVA|NA|F|3M|2003--|USA|TN|	GA5	NA
KJ627731|RSVA|NA|M|1M|2003--|USA|TN|	GA5	NA
KJ627733|RSVA|NA|F|1M|2003--|USA|TN|	GA5	NA
KJ627652|RSVA|NA|M|1M|2002--|USA|TN|	GA5	NA
KJ627674|RSVA|NA|F|1M|2001--|USA|TN|	GA5	NA
KJ627675|RSVA|NA|F|1M|2001--|USA|TN|	GA5	NA
KJ627672|RSVA|NA|F|1M|2001--|USA|TN|	GA5	NA
KJ627710|RSVA|NA|M|5M|2001--|USA|TN|	GA5	NA
KJ627688|RSVA|NA|M|3M|2001--|USA|TN|	GA5	NA
KJ627693|RSVA|NA|F|0.9M|2001--|USA|TN|	GA5	NA
KJ627702|RSVA|NA|F|0.6M|2001--|USA|TN|	GA5	NA

KJ627704|RSVA|NA|F|0.6M|2001--|USA|TN|	GA5	NA
KJ627703|RSVA|NA|F|0.6M|2001--|USA|TN|	GA5	NA
KJ627707|RSVA|NA|F|0.6M|2001--|USA|TN|	GA5	NA
KJ627705|RSVA|NA|F|0.6M|2001--|USA|TN|	GA5	NA
KJ627706|RSVA|NA|F|0.6M|2001--|USA|TN|	GA5	NA
KJ627686|RSVA|NA|M|7M|2001--|USA|TN|	GA5	NA
KJ627687|RSVA|NA|M|7M|2001--|USA|TN|	GA5	NA
KJ627685|RSVA|NA|M|7M|2001--|USA|TN|	GA5	NA
KJ627683|RSVA|NA|F|6M|2001--|USA|TN|	GA5	NA
KJ627684|RSVA|NA|F|6M|2001--|USA|TN|	GA5	NA
KJ627682|RSVA|NA|F|6M|2001--|USA|TN|	GA5	NA
KJ627678|RSVA|NA|F|0.8M|2001--|USA|TN|	GA5	NA
KJ627679|RSVA|NA|F|0.8M|2001--|USA|TN|	GA5	NA
KM360090|RSVA|NA|NA|NA|2001--|USA|TN|	GA5	NA
JX069802|RSVA|NA|M|NA|1998-12-12|USA|TN|	GA5	NA
JX069803|RSVA|NA|M|NA|2000-03-04|USA|TN|	GA5	NA
JX015485|RSVA|GA5|NA|NA|2005-01-04|NLD|NLD|	GA5	NA
JX015488|RSVA|GA5|NA|NA|2006-01-09|NLD|NLD|	GA5	NA
KJ627656|RSVA|NA|F|0.3M|2003--|USA|TN|	GA5	NA
KF530260|RSVA|NA|NA|NA|2005-06-21|ARG|ARG|	GA5	NA
KF826854|RSVA|NA|M|9Y|2009-02-26|ITA|ITA|	GA5	NA
KJ939948|RSVA|NA|NA|NA|2010-07-22|VNM|VNM|	GA5	NA
KJ939954|RSVA|NA|NA|NA|2010-04-20|VNM|VNM|	GA5	NA
KJ939943|RSVA|NA|NA|NA|2010-06-10|VNM|VNM|	GA5	NA
KF826847|RSVA|NA|NA|NA|2007--|AUS|AUS|	GA5	NA
JQ901455|RSVA|GA5|NA|NA|2002-06-29|NLD|NLD|	GA5	NA
KJ627690|RSVA|NA|F|1M|2001--|USA|TN|	GA5	NA
JQ901454|RSVA|GA5|NA|NA|2002-01-04|NLD|NLD|	GA5	NA
KP258727|RSVA|NA|M|1M|1997--|USA|NY|	GA5	NA
KU316180|RSVA|NA|F|2Y|1998--|USA|NY|	GA5	NA
KF826823|RSVA|NA|F|6M|1998-10-17|USA|WI|	GA5	NA
KF826824|RSVA|NA|F|6M|1998-10-28|USA|WI|	GA5	NA
KP258707|RSVA|NA|F|20M|1995--|USA|NY|	GA5	NA
KU316170|RSVA|NA|F|4M|1995--|USA|NY|	GA5	NA
KU316096|RSVA|NA|M|5M|1996--|USA|NY|	GA5	NA
KU316145|RSVA|NA|F|4M|1996--|USA|NY|	GA5	NA
KP258703|RSVA|NA|M|2.5M|1997--|USA|NY|	GA5	NA
KP258726|RSVA|NA|M|13M|1996--|USA|NY|	GA5	NA
KU316176|RSVA|NA|F|5W|1990--|USA|NY|	GA5	NA
KJ723487|RSVA|NA|F|46W|1993--|USA|NY|	GA5	NA

KP258740|RSVA|NA|F|5Y|1993--|USA|NY|	GA5	NA
KU316161|RSVA|NA|F|51D|1993--|USA|NY|	GA5	NA
KP258722|RSVA|NA|F|11M|1993--|USA|NY|	GA5	NA
KP258710|RSVA|NA|M|6Y|1991--|USA|NY|	GA5	NA
KP258732|RSVA|NA|F|18M|1991--|USA|NY|	GA5	NA
KP258728|RSVA|NA|F|9M|1992--|USA|NY|	GA5	NA
KU316104|RSVA|NA|F|9M|1991--|USA|NY|	GA5	NA
KJ723490|RSVA|NA|F|7W|1992--|USA|NY|	GA5	NA
KJ723465|RSVA|NA|F|8W|1992--|USA|NY|	GA5	NA
KP258711|RSVA|NA|M|18Y|1992--|USA|NY|	GA5	NA
KP258701|RSVA|NA|M|18M|1994--|USA|NY|	GA5	NA
KJ723473|RSVA|NA|F|4W|1990--|USA|NY|	GA5	NA
KJ627695|RSVA|NA|F|2M|2001--|USA|TN|	GA5	NA
KJ627696|RSVA|NA|F|2M|2001--|USA|TN|	GA5	NA
KJ723462|RSVA|NA|M|6W|1992--|USA|NY|	GA5	NA
KU316121|RSVA|NA|F|4M|1992--|USA|NY|	GA5	NA
KP258699|RSVA|NA|F|5M|1985--|USA|NY|	GA5	NA
KU316133|RSVA|NA|F|6M|1990--|USA|NY|	GA5	NA
KP258709|RSVA|NA|F|2Y|1981--|USA|NY|	GA5	NA
KU316135|RSVA|NA|F|8W|1981--|USA|NY|	GA5	NA
KJ723488|RSVA|NA|F|4W|1981--|USA|NY|	GA5	NA
KP856967|RSVA|NA|F|1Y|1981--|USA|NY|	GA5	NA
KP258725|RSVA|NA|M|16M|1980--|USA|NY|	GA5	NA
KU316093|RSVA|NA|M|8M|1988--|USA|NY|	GA5	NA
KJ723468|RSVA|NA|F|113W|1988--|USA|NY|	GA5	NA
KP258715|RSVA|NA|M|2Y|1988--|USA|NY|	GA5	NA
KP258696|RSVA|NA|M|22M|1986--|USA|NY|	GA5	NA
KU316150|RSVA|NA|M|5M|1978--|USA|NY|	GA5	NA
KP258704|RSVA|NA|F|2.6M|1994--|USA|NY|	GA5	NA
KU316157|RSVA|NA|F|9W|1979--|USA|NY|	GA5	NA
KU316149|RSVA|NA|M|5M|1977--|USA|NY|	GA5	NA
KU316137|RSVA|NA|F|3Y|1979--|USA|NY|	GA5	NA
KU316178|RSVA|NA|F|12M|1979--|USA|NY|	GA5	NA
KP856969|RSVA|NA|F|1Y|1979--|USA|NY|	GA5	NA
KU316143|RSVA|NA|F|9M|1982--|USA|NY|	GA5	NA
KP258695|RSVA|NA|F|4M|1988--|USA|NY|	GA1	NA
KP258730|RSVA|NA|F|4Y|1987--|USA|NY|	GA1	NA
KU316153|RSVA|NA|F|6M|1988--|USA|NY|	GA1	NA
KU316165|RSVA|NA|M|5W|1987--|USA|NY|	GA1	NA
KP258719|RSVA|NA|F|3Y|1988--|USA|NY|	GA1	NA

KU316162|RSVA|NA|F|22M|1988--|USA|NY|	GA1	NA
KJ723491|RSVA|NA|F|13W|1988--|USA|NY|	GA1	NA
KP258729|RSVA|NA|M|1Y|1990--|USA|NY|	GA1	NA
KP258741|RSVA|NA|M|5M|1990--|USA|NY|	GA1	NA
KU316099|RSVA|NA|M|57D|1997--|USA|NY|	GA1	NA
KU316123|RSVA|NA|M|2M|1997--|USA|NY|	GA1	NA
KP258744|RSVA|NA|F|2Y|1995--|USA|NY|	GA1	NA
KU316164|RSVA|NA|M|4M|1995--|USA|NY|	GA1	NA
KU316107|RSVA|NA|F|3Y|1995--|USA|NY|	GA1	NA
KP258717|RSVA|NA|F|9M|1994--|USA|NY|	GA1	NA
KU316174|RSVA|NA|M|8W|1987--|USA|NY|	GA1	NA
KU316109|RSVA|NA|F|2M|1992--|USA|NY|	GA1	NA
KU316124|RSVA|NA|M|28D|1992--|USA|NY|	GA1	NA
KU316119|RSVA|NA|M|5Y|1989--|USA|NY|	GA1	NA
KJ723489|RSVA|NA|M|29W|1990--|USA|NY|	GA1	NA
KJ723474|RSVA|NA|F|8W|1989--|USA|NY|	GA1	NA
KU316169|RSVA|NA|M|3M|1985--|USA|NY|	GA1	NA
KU316152|RSVA|NA|F|6M|1985--|USA|NY|	GA1	NA
KU316154|RSVA|NA|M|2Y|1985--|USA|NY|	GA1	NA
KJ723461|RSVA|NA|M|2W|1986--|USA|NY|	GA1	NA
KJ723475|RSVA|NA|F|12W|1986--|USA|NY|	GA1	NA
KU316120|RSVA|NA|M|3M|1985--|USA|NY|	GA1	NA
KJ723467|RSVA|NA|M|14W|1986--|USA|NY|	GA1	NA
KU316160|RSVA|NA|F|6M|1986--|USA|NY|	GA1	NA
KU316146|RSVA|NA|M|16M|1983--|USA|NY|	GA1	NA
KJ723478|RSVA|NA|F|4W|1982--|USA|NY|	GA1	NA
KU316103|RSVA|NA|F|2Y|1983--|USA|NY|	GA1	NA
KU316140|RSVA|NA|F|6W|1978--|USA|NY|	GA1	NA
KU316106|RSVA|NA|F|13W|1977--|USA|NY|	GA1	NA
KU316168|RSVA|NA|F|3M|1977--|USA|NY|	GA1	NA
KU316155|RSVA|NA|F|6W|1978--|USA|NY|	GA1	NA
KU316112|RSVA|NA|F|8Y|1980--|USA|NY|	GA1	NA
KU316171|RSVA|NA|F|11M|1977--|USA|NY|	GA1	NA
KU950471_RSVB_NA_NA_NA_2013-08-02_USA_USA_	BA-like	NA
KU950684_RSVB_NA_NA_NA_2013-08-23_USA_USA_	BA-like	NA
KU950591_RSVB_NA_NA_NA_2013-10-01_USA_USA_	BA-like	NA
KU950495_RSVB_NA_NA_NA_2013-07-15_USA_USA_	BA-like	NA
KU839623_RSVB_NA_NA_NA_2013-11-18_USA_TN_	BA-like	NA
KU950481_RSVB_NA_NA_NA_2013-10-01_USA_USA_	BA-like	NA
KU950553_RSVB_NA_NA_NA_2013-08-12_USA_USA_	BA-like	NA

KU950687_RSVB_NA_NA_NA_2013-10-23_USA_USA_	BA-like	NA
KU950593_RSVB_NA_NA_NA_2014-01-09_USA_USA_	BA-like	NA
KU950580_RSVB_NA_NA_NA_2013-10-07_USA_USA_	BA-like	NA
KU839629_RSVB_NA_NA_NA_2013-12-27_USA_TN_	BA-like	NA
KU950532_RSVB_NA_NA_NA_2013-10-07_USA_USA_	BA-like	NA
KU950463_RSVB_NA_NA_NA_2013-11-26_USA_USA_	BA-like	NA
KU839628_RSVB_NA_NA_NA_2013-11-27_USA_TN_	BA-like	NA
KU950675_RSVB_NA_NA_NA_2013-06-21_USA_USA_	BA-like	NA
KU950676_RSVB_NA_NA_NA_2014-01-09_USA_USA_	BA-like	NA
KU950582_RSVB_NA_NA_NA_2013-07-17_USA_USA_	BA-like	NA
KU950534_RSVB_NA_NA_NA_2014-01-15_USA_USA_	BA-like	NA
KU839634_RSVB_NA_NA_NA_2013-11-15_USA_TN_	BA-like	NA
KU950681_RSVB_NA_NA_NA_2013-12-05_USA_USA_	BA-like	NA
KU950678_RSVB_NA_NA_NA_2013-08-23_USA_USA_	BA-like	NA
KU950482_RSVB_NA_NA_NA_2013-08-05_USA_USA_	BA-like	NA
KU950565_RSVB_NA_NA_NA_2013-08-16_USA_USA_	BA-like	NA
KU950462_RSVB_NA_NA_NA_2013-08-26_USA_USA_	BA-like	NA
KU950512_RSVB_NA_NA_NA_2013-07-10_USA_USA_	BA-like	NA
KU950525_RSVB_NA_NA_NA_2014-03-20_USA_USA_	BA-like	NA
KU950559_RSVB_NA_NA_NA_2013-07-22_USA_USA_	BA-like	NA
KU950577_RSVB_NA_NA_NA_2013-11-19_USA_USA_	BA-like	NA
KU950659_RSVB_NA_NA_NA_2013-11-20_USA_USA_	BA-like	NA
KU950516_RSVB_NA_NA_NA_2013-08-15_USA_USA_	BA-like	NA
KU950466_RSVB_NA_NA_NA_2013-10-17_USA_USA_	BA-like	NA
KU839640_RSVB_NA_NA_NA_2013-12-11_USA_TN_	BA-like	NA
KU950515_RSVB_NA_NA_NA_2013-10-14_USA_USA_	BA-like	NA
KU950476_RSVB_NA_NA_NA_2013-10-08_USA_USA_	BA-like	NA
KU950691_RSVB_NA_NA_NA_2013-06-19_USA_USA_	BA-like	NA
KU950695_RSVB_NA_NA_NA_2013-07-30_USA_USA_	BA-like	NA
KU839635_RSVB_NA_NA_NA_2013-12-02_USA_TN_	BA-like	NA
KU950504_RSVB_NA_NA_NA_2013-06-18_USA_USA_	BA-like	NA
KU950589_RSVB_NA_NA_NA_2013-10-29_USA_USA_	BA-like	NA
KU950605_RSVB_NA_NA_NA_2013-08-13_USA_USA_	BA-like	NA
KU950656_RSVB_NA_NA_NA_2013-09-16_USA_USA_	BA-like	NA
KU839627_RSVB_NA_NA_NA_2014-01-29_USA_TN_	BA-like	NA
KU950568_RSVB_NA_NA_NA_2014-01-07_USA_USA_	BA-like	NA
KU950517_RSVB_NA_NA_NA_2013-10-22_USA_USA_	BA-like	NA
KU950690_RSVB_NA_NA_NA_2013-08-14_USA_USA_	BA-like	NA
KU839632_RSVB_NA_NA_NA_2013-12-17_USA_TN_	BA-like	NA
KU950510_RSVB_NA_NA_NA_2013-09-25_USA_USA_	BA-like	NA

KU950503_RSVB_NA_NA_NA_2013-11-15_USA_USA_	BA-like	NA
KU950648_RSVB_NA_NA_NA_2014-01-27_USA_USA_	BA-like	NA
KU950672_RSVB_NA_NA_NA_2013-12-09_USA_USA_	BA-like	NA
KU950697_RSVB_NA_NA_NA_2013-12-16_USA_USA_	BA-like	NA
KU950688_RSVB_NA_NA_NA_2013-08-28_USA_USA_	BA-like	NA
KU950562_RSVB_NA_NA_NA_2013-09-23_USA_USA_	BA-like	NA
KU950665_RSVB_NA_NA_NA_2013-11-22_USA_USA_	BA-like	NA
KU950514_RSVB_NA_NA_NA_2013-11-21_USA_USA_	BA-like	NA
KU839641_RSVB_NA_NA_NA_2014-01-20_USA_TN_	BA-like	NA
KU950465_RSVB_NA_NA_NA_2013-11-19_USA_USA_	BA-like	NA
KU950497_RSVB_NA_NA_NA_2013-08-20_USA_USA_	BA-like	NA
KU950630_RSVB_NA_NA_NA_2013-07-05_USA_USA_	BA-like	NA
KU950668_RSVB_NA_NA_NA_2013-09-30_USA_USA_	BA-like	NA
KU950679_RSVB_NA_NA_NA_2013-07-12_USA_USA_	BA-like	NA
KU950555_RSVB_NA_NA_NA_2013-12-03_USA_USA_	BA-like	NA
KU839638_RSVB_NA_NA_NA_2013-11-11_USA_TN_	BA-like	NA
KU950663_RSVB_NA_NA_NA_2013-10-08_USA_USA_	BA-like	NA
KU950599_RSVB_NA_NA_NA_2013-10-29_USA_USA_	BA-like	NA
KU950601_RSVB_NA_NA_NA_2013-10-23_USA_USA_	BA-like	NA
KU950622_RSVB_NA_NA_NA_2013-10-15_USA_USA_	BA-like	NA
KU950478_RSVB_NA_NA_NA_2013-06-20_USA_USA_	BA-like	NA
KU950539_RSVB_NA_NA_NA_2013-09-25_USA_USA_	BA-like	NA
KU950543_RSVB_NA_NA_NA_2013-10-16_USA_USA_	BA-like	NA
KU950606_RSVB_NA_NA_NA_2013-12-13_USA_USA_	BA-like	NA
KU950689_RSVB_NA_NA_NA_2013-10-31_USA_USA_	BA-like	NA
KU950526_RSVB_NA_NA_NA_2013-11-27_USA_USA_	BA-like	NA
KU950535_RSVB_NA_NA_NA_2013-06-25_USA_USA_	BA-like	NA
KU950496_RSVB_NA_NA_NA_2013-12-02_USA_USA_	BA-like	NA
KU950578_RSVB_NA_NA_NA_2013-10-23_USA_USA_	BA-like	NA
KU950494_RSVB_NA_NA_NA_2013-12-03_USA_USA_	BA-like	NA
KU950530_RSVB_NA_NA_NA_2014-01-21_USA_USA_	BA-like	NA
KU950477_RSVB_NA_NA_NA_2014-01-27_USA_USA_	BA-like	NA
KU950552_RSVB_NA_NA_NA_2013-12-27_USA_USA_	BA-like	NA
KU950613_RSVB_NA_NA_NA_2013-09-27_USA_USA_	BA-like	NA
KU950618_RSVB_NA_NA_NA_2012-10-17_USA_USA_	BA-like	NA
JX576734_RSVB_GB13_NA_NA_2012-01-29_NLD_NLD_	BA-like	NA
JX576736_RSVB_GB13_NA_NA_2012-01-07_NLD_NLD_	BA-like	NA
61945_RSVB_NA_NA_NA_2013-01-06_JOR_Amman_	BA-like	5
62012_RSVB_NA_NA_NA_2011-02-13_JOR_Amman_	BA-like	5
62009_RSVB_NA_NA_NA_2013-03-17_JOR_Amman_	BA-like	14

61948_RSVB_NA_NA_NA_2013-02-10_JOR_Amman_	BA-like	9
61949_RSVB_NA_NA_NA_2013-02-11_JOR_Amman_	BA-like	9
61940_RSVB_NA_NA_NA_2013-02-20_JOR_Amman_	BA-like	9
61942_RSVB_NA_NA_NA_2012-12-30_JOR_Amman_	BA-like	9
61941_RSVB_NA_NA_NA_2013-03-03_JOR_Amman_	BA-like	9
61950_RSVB_NA_NA_NA_2013-02-14_JOR_Amman_	BA-like	9
61938_RSVB_NA_NA_NA_2013-02-21_JOR_Amman_	BA-like	9
62008_RSVB_NA_NA_NA_2013-03-27_JOR_Amman_	BA-like	9
61953_RSVB_NA_NA_NA_2013-01-17_JOR_Amman_	BA-like	9
KU950488_RSVB_NA_NA_NA_2014-01-25_USA_USA_	BA-like	NA
JX576735_RSVB_GB13_NA_NA_2012-01-23_NLD_NLD_	BA-like	NA
KU950669_RSVB_NA_NA_NA_2013-07-09_USA_USA_	BA-like	NA
61967_RSVB_NA_NA_NA_2010-03-24_JOR_Amman_	BA-like	1
KU950621_RSVB_NA_NA_NA_2014-02-05_USA_USA_	BA-like	NA
KU950558_RSVB_NA_NA_NA_2014-01-08_USA_USA_	BA-like	NA
KU950461_RSVB_NA_NA_NA_2013-12-17_USA_USA_	BA-like	NA
61939_RSVB_NA_NA_NA_2013-02-26_JOR_Amman_	BA-like	13
KU950533_RSVB_NA_NA_NA_2014-01-10_USA_USA_	BA-like	NA
KU839633_RSVB_NA_NA_NA_2014-01-13_USA_TN_	BA-like	NA
KU950542_RSVB_NA_NA_NA_2013-10-07_USA_USA_	BA-like	NA
KU950588_RSVB_NA_NA_NA_2014-01-22_USA_USA_	BA-like	NA
KU950508_RSVB_NA_NA_NA_2013-07-30_USA_USA_	BA-like	NA
KU950547_RSVB_NA_NA_NA_2013-11-19_USA_USA_	BA-like	NA
KU950548_RSVB_NA_NA_NA_2014-01-24_USA_USA_	BA-like	NA
KU950571_RSVB_NA_NA_NA_2012-12-19_USA_USA_	BA-like	NA
KJ672476_RSVB_NA_F_3M_2013-01-04_USA_TN_	BA-like	NA
KU950586_RSVB_NA_NA_NA_2013-01-09_USA_USA_	BA-like	NA
KU950633_RSVB_NA_NA_NA_2013-12-10_USA_USA_	BA-like	NA
JX576741_RSVB_GB13_NA_NA_2009-12-22_NLD_NLD_	BA-like	NA
61964_RSVB_NA_NA_NA_2010-03-21_JOR_Amman_	BA-like	NA
KJ627262_RSVB_NA_M_1Y_2012-03-14_PER_PER_	BA-like	NA
KJ627342_RSVB_NA_F_4M_2012-02-23_PER_PER_	BA-like	NA
KJ627340_RSVB_NA_F_4M_2012-02-28_PER_PER_	BA-like	NA
JX576737_RSVB_GB13_NA_NA_2010-12-21_NLD_NLD_	BA-like	NA
JX576733_RSVB_GB13_NA_NA_2012-02-05_NLD_NLD_	BA-like	NA
KF826858_RSVB_NA_F_2M_2009-03-04_ITA_ITA_	BA-like	NA
KP317928_RSVB_NA_NA_NA_2011-07-08_KEN_KEN_	BA-like	NA
KJ939923_RSVB_NA_NA_NA_2009-10-07_VNM_VNM_	BA-like	NA
KJ939927_RSVB_NA_NA_NA_2010-05-27_VNM_VNM_	BA-like	NA
KJ939934_RSVB_NA_NA_NA_2010-09-08_VNM_VNM_	BA-like	NA

KJ939921_RSVB_NA_NA_NA_2009-08-31_VNM_VNM_	BA-like	NA
KJ939925_RSVB_NA_NA_NA_2009-11-18_VNM_VNM_	BA-like	NA
KJ939931_RSVB_NA_NA_NA_2009-11-10_VNM_VNM_	BA-like	NA
KJ939924_RSVB_NA_NA_NA_2009-10-23_VNM_VNM_	BA-like	NA
61956_RSVB_NA_NA_NA_2012-12-19_JOR_Amman_	BA-like	7
62010_RSVB_NA_NA_NA_2013-03-13_JOR_Amman_	BA-like	7
KR350475_RSVB_NA_NA_NA_2014-11-21_MEX_MEX_	BA-like	NA
KU950584_RSVB_NA_NA_NA_2013-10-31_USA_USA_	BA-like	NA
KJ939928_RSVB_NA_NA_NA_2010-06-17_VNM_VNM_	BA-like	NA
JX576738_RSVB_GB13_NA_NA_2010-12-12_NLD_NLD_	BA-like	NA
JX576745_RSVB_GB13_NA_NA_2008-12-17_NLD_NLD_	BA-like	NA
JX576732_RSVB_GB13_NA_NA_2006-12-05_BEL_BEL_	BA-like	NA
61931_RSVB_NA_NA_NA_2011-01-31_JOR_Amman_	BA-like	4
61992_RSVB_NA_NA_NA_2012-04-05_JOR_Amman_	BA-like	4
KU950587_RSVB_NA_NA_NA_2013-11-13_USA_USA_	BA-like	NA
KU950604_RSVB_NA_NA_NA_2013-06-19_USA_USA_	BA-like	NA
KU950635_RSVB_NA_NA_NA_2013-11-18_USA_USA_	BA-like	NA
KU950637_RSVB_NA_NA_NA_2014-02-04_USA_USA_	BA-like	NA
61958_RSVB_NA_NA_NA_2013-02-05_JOR_Amman_	BA-like	12
KU839625_RSVB_NA_NA_NA_2014-01-02_USA_TN_	BA-like	NA
KU950607_RSVB_NA_NA_NA_2013-12-06_USA_USA_	BA-like	NA
61952_RSVB_NA_NA_NA_2013-01-15_JOR_Amman_	BA-like	10
62006_RSVB_NA_NA_NA_2013-03-13_JOR_Amman_	BA-like	10
KU950467_RSVB_NA_NA_NA_2014-01-15_USA_USA_	BA-like	NA
KU950611_RSVB_NA_NA_NA_2013-10-14_USA_USA_	BA-like	NA
KU950682_RSVB_NA_NA_NA_2012-08-15_USA_USA_	BA-like	NA
KP663730_RSVB_NA_NA_NA_2014-01-13_KOR_KOR_	BA-like	NA
KM517573_RSVB_NA_NA_NA_2013-07-30_CHN_GZ_	BA-like	NA
KP663729_RSVB_NA_NA_NA_2014-01-14_KOR_KOR_	BA-like	NA
KU950603_RSVB_NA_NA_NA_2013-09-19_USA_USA_	BA-like	NA
61959_RSVB_NA_NA_NA_2013-01-31_JOR_Amman_	BA-like	11
61957_RSVB_NA_NA_NA_2012-12-27_JOR_Amman_	BA-like	8
KJ939929_RSVB_NA_NA_NA_2010-07-07_VNM_VNM_	BA-like	NA
JX576749_RSVB_GB13_NA_NA_2007-11-23_NLD_NLD_	BA-like	NA
JX576740_RSVB_GB13_NA_NA_2009-12-28_NLD_NLD_	BA-like	NA
JX576743_RSVB_GB13_NA_NA_2009-12-18_NLD_NLD_	BA-like	NA
KU950458_RSVB_NA_NA_NA_2013-12-16_USA_USA_	BA-like	NA
KU950569_RSVB_NA_NA_NA_2013-03-04_USA_USA_	BA-like	NA
KJ627359_RSVB_NA_M_9M_2011-04-13_PER_PER_	BA-like	NA
KJ939920_RSVB_NA_NA_NA_2009-08-17_VNM_VNM_	BA-like	NA

KF826851_RSVB_NA_NA_NA_2007_USA_WI_	BA-like	NA
KJ627364_RSVB_NA_F_10M_2011-05-12_PER_PER_	BA-like	NA
KJ627254_RSVB_NA_M_1Y_2011-06-13_PER_PER_	BA-like	NA
KF826857_RSVB_NA_M_4M_2009-02-18_ITA_ITA_	BA-like	NA
KJ627348_RSVB_NA_F_1Y_2011-06-02_PER_PER_	BA-like	NA
JX576744_RSVB_GB13_NA_NA_2008-12-22_NLD_NLD_	BA-like	NA
JX576748_RSVB_GB13_NA_NA_2007-11-27_NLD_NLD_	BA-like	NA
KJ627278_RSVB_NA_M_5M_2010-03-10_PER_PER_	BA-like	NA
KJ627341_RSVB_NA_M_7M_2011-04-04_PER_PER_	BA-like	NA
KJ627330_RSVB_NA_F_1Y_2008-07-15_PER_PER_	BA-like	NA
JX576747_RSVB_GB13_NA_NA_2008-12-12_NLD_NLD_	BA-like	NA
KF826820_RSVB_NA_M_1M_2009-03-31_ITA_ITA_	BA-like	NA
KF826860_RSVB_NA_F_10M_2009-04-28_ITA_ITA_	BA-like	NA
KF826859_RSVB_NA_M_3M_2009-03-05_ITA_ITA_	BA-like	NA
JX576730_RSVB_GB13_NA_NA_2008-10-24_BEL_BEL_	BA-like	NA
JX576729_RSVB_GB13_NA_NA_2008-10-23_BEL_BEL_	BA-like	NA
JX576731_RSVB_GB13_NA_NA_2008-11-19_BEL_BEL_	BA-like	NA
JX576746_RSVB_GB13_NA_NA_2008-12-15_NLD_NLD_	BA-like	NA
KF826822_RSVB_NA_NA_NA_2007_USA_WI_	BA-like	NA
JX576751_RSVB_GB13_NA_NA_2007-01-05_NLD_NLD_	BA-like	NA
JX576753_RSVB_GB13_NA_NA_2006-11-17_NLD_NLD_	BA-like	NA
JN032120_RSVB_NA_NA_NA_2010-01-21_USA_WI_	BA-like	NA
KJ627285_RSVB_NA_F_8M_2011-06-09_PER_PER_	BA-like	NA
KP317927_RSVB_NA_NA_NA_2011-02-01_KEN_KEN_	BA-like	NA
KP317945_RSVB_NA_NA_NA_2011-04-14_KEN_KEN_	BA-like	NA
KP317934_RSVB_NA_NA_NA_2012-02-13_KEN_KEN_	BA-like	NA
KP317952_RSVB_NA_NA_NA_2010-05-09_KEN_KEN_	BA-like	NA
KP317917_RSVB_NA_NA_NA_2006-01-27_KEN_KEN_	BA-like	NA
KP317932_RSVB_NA_NA_NA_2010-04-06_KEN_KEN_	BA-like	NA
KP317941_RSVB_NA_NA_NA_2010-02-13_KEN_KEN_	BA-like	NA
JX576756_RSVB_GB13_NA_NA_2005-12-20_NLD_NLD_	BA-like	NA
KF826842_RSVB_NA_NA_NA_2007-05-22_ARG_ARG_	BA-like	NA
KF530259_RSVB_NA_NA_NA_2006_ZAF_ZAF_	BA-like	NA
KJ627280_RSVB_NA_F_1Y_2008-02-11_PER_PER_	BA-like	NA
KJ627317_RSVB_NA_M_9M_2007-01-29_PER_PER_	BA-like	NA
KJ627310_RSVB_NA_F_2Y_2007-01-31_PER_PER_	BA-like	NA
KJ627299_RSVB_NA_F_1Y_2007-11-24_PER_PER_	BA-like	NA
JX576750_RSVB_GB13_NA_NA_2007-03-14_NLD_NLD_	BA-like	NA
KJ939930_RSVB_NA_NA_NA_2009-10-26_VNM_VNM_	BA-like	NA
JX576752_RSVB_GB13_NA_NA_2006-12-21_NLD_NLD_	BA-like	NA

JX576757_RSVB_GB13_NA_NA_2005-01-14_NLD_NLD_	BA-like	NA
KP317925_RSVB_NA_NA_NA_2004-12-22_KEN_KEN_	BA-like	NA
KU950574_RSVB_NA_NA_NA_2005-12-03_USA_USA_	BA-like	NA
KU950614_RSVB_NA_NA_NA_2005-12-29_USA_USA_	BA-like	NA
KU950457_RSVB_NA_NA_NA_2005-12-30_USA_USA_	BA-like	NA
JX576754_RSVB_GB13_NA_NA_2005-12-31_NLD_NLD_	BA-like	NA
KJ627302_RSVB_NA_F_9M_2008-04-12_PER_PER_	BA-like	NA
KF826839_RSVB_NA_NA_NA_2006-07-25_ARG_ARG_	BA-like	NA
JX576759_RSVB_GB13_NA_NA_2003-12-05_NLD_NLD_	BA-like	NA
JX576755_RSVB_GB13_NA_NA_2005-12-26_NLD_NLD_	BA-like	NA
KJ672425_RSVB_NA_M_4M_2013-01-28_USA_TN_	BA-like	NA
KU950500_RSVB_NA_NA_NA_2012-12-18_USA_USA_	BA-like	NA
KU950576_RSVB_NA_NA_NA_2012-11-02_USA_USA_	BA-like	NA
KM042393_RSVB_NA_NA_NA_2012-11-26_USA_TN_	BA-like	NA
KU950619_RSVB_NA_NA_NA_2012-08-27_USA_USA_	BA-like	NA
KJ672438_RSVB_NA_F_3M_2013-01-21_USA_TN_	BA-like	NA
KJ672430_RSVB_NA_M_3M_2013-01-18_USA_TN_	BA-like	NA
KJ672481_RSVB_NA_F_4M_2013-02-26_USA_TN_	BA-like	NA
KU950693_RSVB_NA_NA_NA_2012-10-16_USA_USA_	BA-like	NA
KU839636_RSVB_NA_NA_NA_2013-12-27_USA_TN_	BA-like	NA
KU950484_RSVB_NA_NA_NA_2013-12-09_USA_USA_	BA-like	NA
KU950625_RSVB_NA_NA_NA_2014-01-14_USA_USA_	BA-like	NA
KU950489_RSVB_NA_NA_NA_2013-06-24_USA_USA_	BA-like	NA
KU950647_RSVB_NA_NA_NA_2012-12-04_USA_USA_	BA-like	NA
KU950662_RSVB_NA_NA_NA_2012-12-14_USA_USA_	BA-like	NA
KU950602_RSVB_NA_NA_NA_2013-11-27_USA_USA_	BA-like	NA
62003_RSVB_NA_NA_NA_2012-04-24_JOR_Amman_	BA-like	6
61966_RSVB_NA_NA_NA_2010-03-31_JOR_Amman_	BA-like	2
KJ939926_RSVB_NA_NA_NA_2010-05-05_VNM_VNM_	BA-like	NA
JX576739_RSVB_GB13_NA_NA_2009-12-28_NLD_NLD_	BA-like	NA
KU950657_RSVB_NA_NA_NA_2012-08-29_USA_USA_	BA-like	NA
KF826845_RSVB_NA_NA_NA_2008-06-10_ARG_ARG_	BA-like	NA
KF826843_RSVB_NA_M_8M_2008-09-07_MEX_MEX_	BA-like	NA
KF826844_RSVB_NA_M_5M_2008-10-19_MEX_MEX_	BA-like	NA
JX576742_RSVB_GB13_NA_NA_2009-12-20_NLD_NLD_	BA-like	NA
KJ627247_RSVB_NA_M_11M_2011-06-03_PER_PER_	BA-like	NA
KJ627332_RSVB_NA_F_8M_2011-06-13_PER_PER_	BA-like	NA
KJ627277_RSVB_NA_M_1Y_2011-05-19_PER_PER_	BA-like	NA
61981_RSVB_NA_NA_NA_2010-06-27_JOR_Amman_	BA-like	3
JX576761_RSVB_GB13_NA_NA_2002-10-21_NLD_NLD_	BA-like	NA

KF826825_RSVB_NA_F_5M_2004-10-18_MEX_MEX_	BA-like	NA
KJ939919_RSVB_NA_NA_NA_2009-07-30_VNM_VNM_	BA-like	NA
KJ939922_RSVB_NA_NA_NA_2009-09-24_VNM_VNM_	BA-like	NA
JX576758_RSVB_GB13_NA_NA_2005-12-14_NLD_NLD_	BA-like	NA
JX576762_RSVB_GB13_NA_NA_2002-01-08_NLD_NLD_	BA-like	NA
KF826829_RSVB_NA_F_30M_2005-12-13_MEX_MEX_	BA-like	NA
JQ582844_RSVB_NA_NA_NA_2002_USA_USA_	BA-like	NA
JQ582843_RSVB_NA_NA_NA_2002_USA_USA_	Non BA-like	NA
JX576760_RSVB_GB12_NA_NA_2003-01-01_NLD_NLD_	Non BA-like	NA
KP258724_RSVB_NA_NA_NA_1997_USA_NY_	Non BA-like	NA
KU316172_RSVB_NA_NA_NA_1997_USA_NY_	Non BA-like	NA
KP856966_RSVB_NA_NA_NA_1998_USA_NY_	Non BA-like	NA
KU316100_RSVB_NA_NA_NA_1994_USA_NY_	Non BA-like	NA
KU316105_RSVB_NA_NA_NA_1998_USA_NY_	Non BA-like	NA
KU316158_RSVB_NA_NA_NA_1996_USA_NY_	Non BA-like	NA
KP258742_RSVB_NA_NA_NA_1994_USA_NY_	Non BA-like	NA
KU316134_RSVB_NA_NA_NA_1994_USA_NY_	Non BA-like	NA
KP258739_RSVB_NA_NA_NA_1996_USA_NY_	Non BA-like	NA
KP258708_RSVB_NA_NA_NA_1996_USA_NY_	Non BA-like	NA
KP258713_RSVB_NA_NA_NA_1993_USA_NY_	Non BA-like	NA
KP258745_RSVB_NA_NA_NA_1992_USA_NY_	Non BA-like	NA
KF826853_RSVB_NA_NA_NA_2008-02-01_DEU_DEU_	Non BA-like	NA
KP317923_RSVB_NA_NA_NA_2012-03-01_KEN_KEN_	Non BA-like	NA
KU316179_RSVB_NA_NA_NA_1994_USA_NY_	Non BA-like	NA
KP856961_RSVB_NA_NA_NA_1989_USA_NY_	Non BA-like	NA
KP856965_RSVB_NA_NA_NA_1989_USA_NY_	Non BA-like	NA
KP258705_RSVB_NA_NA_NA_1995_USA_NY_	Non BA-like	NA
KU316117_RSVB_NA_NA_NA_1995_USA_NY_	Non BA-like	NA
KP258702_RSVB_NA_NA_NA_1994_USA_NY_	Non BA-like	NA
KJ723484_RSVB_NA_M_10W_1993_USA_NY_	Non BA-like	NA
KU316114_RSVB_NA_NA_NA_1993_USA_NY_	Non BA-like	NA
KU316128_RSVB_NA_NA_NA_1995_USA_NY_	Non BA-like	NA
KU316159_RSVB_NA_NA_NA_1997_USA_NY_	Non BA-like	NA
KU316156_RSVB_NA_NA_NA_1989_USA_NY_	Non BA-like	NA
KJ723466_RSVB_NA_F_5W_1993_USA_NY_	Non BA-like	NA
KU316163_RSVB_NA_NA_NA_1993_USA_NY_	Non BA-like	NA
KU316111_RSVB_NA_NA_NA_1992_USA_NY_	Non BA-like	NA
KP258720_RSVB_NA_NA_NA_1990_USA_NY_	Non BA-like	NA
KU316132_RSVB_NA_NA_NA_1991_USA_NY_	Non BA-like	NA
KU316144_RSVB_NA_NA_NA_1991_USA_NY_	Non BA-like	NA

KU316127_RSVB_NA_NA_NA_1991_USA_NY_	Non BA-like	NA
KJ723480_RSVB_NA_M_9W_1991_USA_NY_	Non BA-like	NA
KJ723470_RSVB_NA_M_18W_1991_USA_NY_	Non BA-like	NA
KP258698_RSVB_NA_NA_NA_1991_USA_NY_	Non BA-like	NA
KJ723477_RSVB_NA_M_21W_1991_USA_NY_	Non BA-like	NA
KJ723460_RSVB_NA_M_5W_1991_USA_NY_	Non BA-like	NA
KJ723482_RSVB_NA_M_15W_1989_USA_NY_	Non BA-like	NA
KU316113_RSVB_NA_NA_NA_1989_USA_NY_	Non BA-like	NA
KU316181_RSVB_NA_NA_NA_1990_USA_NY_	Non BA-like	NA
KP258714_RSVB_NA_NA_NA_1986_USA_NY_	Non BA-like	NA
KU316182_RSVB_NA_NA_NA_1990_USA_NY_	Non BA-like	NA
KU316102_RSVB_NA_NA_NA_1982_USA_NY_	Non BA-like	NA
KU316175_RSVB_NA_NA_NA_1985_USA_NY_	Non BA-like	NA
KJ723481_RSVB_NA_M_8W_1985_USA_NY_	Non BA-like	NA
KU316130_RSVB_NA_NA_NA_1985_USA_NY_	Non BA-like	NA
KU316173_RSVB_NA_NA_NA_1984_USA_NY_	Non BA-like	NA
KP258735_RSVB_NA_NA_NA_1987_USA_NY_	Non BA-like	NA
KJ723463_RSVB_NA_F_5W_1987_USA_NY_	Non BA-like	NA
KU316108_RSVB_NA_NA_NA_1987_USA_NY_	Non BA-like	NA
KJ723469_RSVB_NA_M_18W_1987_USA_NY_	Non BA-like	NA
KU316129_RSVB_NA_NA_NA_1987_USA_NY_	Non BA-like	NA
KU316151_RSVB_NA_NA_NA_1986_USA_NY_	Non BA-like	NA
KJ723476_RSVB_NA_M_9W_1987_USA_NY_	Non BA-like	NA
KP258731_RSVB_NA_NA_NA_1982_USA_NY_	Non BA-like	NA
KP258721_RSVB_NA_NA_NA_1981_USA_NY_	Non BA-like	NA
KP258712_RSVB_NA_NA_NA_1979_USA_NY_	Non BA-like	NA
KP258736_RSVB_NA_NA_NA_1982_USA_NY_	Non BA-like	NA
KU316177_RSVB_NA_NA_NA_1982_USA_NY_	Non BA-like	NA
KU316101_RSVB_NA_NA_NA_1982_USA_NY_	Non BA-like	NA
KJ723479_RSVB_NA_M_5W_1983_USA_NY_	Non BA-like	NA
KP258718_RSVB_NA_NA_NA_1983_USA_NY_	Non BA-like	NA
KP856963_RSVB_NA_NA_NA_1980_USA_NY_	Non BA-like	NA
KU316122_RSVB_NA_NA_NA_1980_USA_NY_	Non BA-like	NA
KU316116_RSVB_NA_NA_NA_1977_USA_NY_	Non BA-like	NA
KU316095_RSVB_NA_NA_NA_1979_USA_NY_	Non BA-like	NA
KU316147_RSVB_NA_NA_NA_1979_USA_NY_	Non BA-like	NA
KJ723485_RSVB_NA_F_13W_1984_USA_NY_	Non BA-like	NA
KU316136_RSVB_NA_NA_NA_1987_USA_NY_	Non BA-like	NA
KP258738_RSVB_NA_NA_NA_1980_USA_NY_	Non BA-like	NA
KU316115_RSVB_NA_NA_NA_1980_USA_NY_	Non BA-like	NA
